# Novel Anthraquinone Chlorination Contributes to Pigmentation and ATP Formation in *Thermomyces dupontii*

**DOI:** 10.1111/1751-7915.70254

**Published:** 2025-10-22

**Authors:** Donglou Wang, Gang Dai, Jiangbo He, Huiwen Si, Chunhua Liao, Wenjie Wang, Zhangxin Zuo, Shuhong Li, Xuemei Niu

**Affiliations:** ^1^ State Key Laboratory for Conservation and Utilization of Bio‐Resources in Yunnan andYunnan Key Laboratory of Basic Research and Innovative Application for Green Biological Production, Key Laboratory for Microbial Resources of the Ministry of Education, School of Life Sciences Yunnan University Kunming P. R. China; ^2^ Kunming Key Laboratory of Respiratory Disease Kunming University Kunming P. R. China

**Keywords:** anthraquinones, ATP, dichlorinated anthraquinones, halogenase gene, pigmentation, *Thermomyces dupontii*, thermophilic fungus

## Abstract

Fungal pigments, particularly anthraquinones, play critical roles in food industrial applications due to their vivid hues and bioactive properties. Despite their significance, the functional mechanisms underlying pigment variation and their natural functions remain poorly understood. Here, we investigate the genetic and biochemical basis of anthraquinone‐mediated pigmentation in the thermophilic fungus *Thermomyces dupontii*. Through a combination of transcriptomic, biochemical and chemical analyses, we identified the flavin‐dependent halogenase gene (*hal*) as a key controller of anthraquinone chlorination, a process essential for fungal pigmentation and metabolic responses to low temperature stress. Disruption of *hal* abolished chlorinated anthraquinone production, reduced colony pigmentation and impaired ATP production. We characterised novel chlorinated and dichlorinated anthraquinones, highlighting chlorination's role in enhancing the structural diversity of anthraquinones and fungal pigmentation. Detailed bioassays indicated that *hal*‐mediated anthraquinone chlorination contributes to fungal survival under cold stress by enhancing two distinct energy modes. This study provides new insights into the ecological significance of fungal pigmentation and opens avenues for the biotechnological exploitation of fungal pigments in food industrial and medical research.

## Graphical abstract

This diagram illustrates the chlorination of anthraquinones and its biological functions in *Thermomyces dupontii*, elucidating how chlorination‐mediated fungal pigmentation promotes energy production and stress adaptation. (1) Extracellular Fenton Chemistry: Involves superoxide anion (O_2_^−^) and ferrous ion (Fe^2+^), which are two fundamental elements for extracellular Fenton chemistry, which is strongly enhanced by polyketide‐derived emodin (5) and carviolin A (1) that facilitate the formation of Fe^2+^ and O_2_^−^ and export Fe^2+^. The extracellular Fenton chemistry can degrade organic matter to generate energy and elevate the ambient temperatures, collectively enabling fungal adaptation to low‐temperature conditions. (2) Anthraquinone Chlorination: Halogenase (Hal) in *T. dupontii* can introduce chlorine (Cl) to emodin and carviolin A, generating yellow monochlorinated intermediates: carviolin A‐Cl (2) and emodin‐Cl (4). These two intermediates were further fused to form a new dark purple dimeric anthraquinone. (3) Linkage to Two Energy Modes: The chlorination of anthraquinones enhances intracellular ATP production and extracellular Fenton chemistry via releasing protons and electrons from anthraquinone and Cl^−^, thereby contributing to fungal colour and vitality under low temperature stress. In summary, this figure illustrates how *T. dupontii* employs anthraquinone chlorination (driven by the *hal* gene) to enhance pigmentation, energy production and cold stress tolerance. This connection bridges chemical transformations with ecological adaptation, highlighting the biotechnological potential of fungal pigments in environmental ecology research.

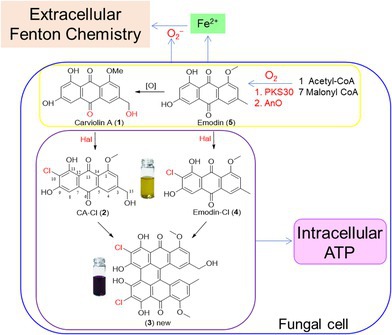

## 1 Introduction

Coloration is one of the most important functional traits on earth, with versatile roles as a visual signal and as pigments (Cuthill et al. 2017). Through coloration, organisms interact with their environment and convey crucial information about individual and species identity, health status and community dominance (Amundsen 2000; Doucet and Meadows 2009; Cuthill et al. 2017; Mason and Bowie 2020; Arbore et al. 2024). Fungi, one of the largest groups of organisms on Earth (Tedersoo et al. 2014; Hawksworth and Lücking 2017; Niskanen et al. 2023), are essential for carbon and nutrient cycling, thereby contributing to ecosystem stability (Treseder and Lennon 2015; Grossart et al. 2019; Coleine et al. 2022; Liu et al. 2022). The production of pigments by fungal colonies has attracted interest since the 19th century and is recognised as a microbial reserve for producing food‐grade pigments (Kalra et al. 2020). Fungal pigments, known for their vivid hues and diverse patterns, provide a sustainable alternative to synthetic dyes in food colourants and cosmetics due to their natural origin and biodegradability (Durán et al. 2002; De Oliveira et al. 2024; Díez et al. 2025). These pigments are biosynthesized within the cytoplasm in response to adverse ecological conditions, fulfilling ecological functions. However, the mechanism of the colour variability within fungal species and its natural ecological functions still remains largely unexploited (Hari et al. 1994; Gmoser et al. 2017; Sajjad et al. 2020).

Anthraquinones are among the most common quinoid natural pigments found in nature, occurring in plants, fungi, lichens, algae, microorganisms, invertebrates and vertebrate animals (Hari et al. 1994; Dufossé 2014; Li, Wang, et al. 2024). These compounds consist of a linear three‐ring quinone structure, typically exhibiting yellow, orange, or brown pigments (Shahid et al. 2019; Do et al. 2022). The anthraquinone scaffold has been extensively studied for its application in food colourants, cosmetics, and pharmacological properties. Historically, anthraquinones, such as aloe‐emodin, have been used as food additives for many centuries (Baqi 2016; Malik and Müller 2016; Catalano et al. 2024; Luo et al. 2024). Moreover, anthraquinones have been shown to inhibit enzymes such as topoisomerases, telomerase, protein kinases, matrix metalloproteinases (Zn and Ca‐dependent neutral endopeptidases) and ecto‐nucleotidases (Malik and Müller 2016; Tian et al. 2020; Li, Wang, et al. 2024). However, few studies have investigated variation in the hue of anthraquinone‐based pigments in nature or the functional mechanisms that facilitate host adaptation to environmental changes (Davies et al. 2022; Gessler et al. 2013).

Anthraquinones are generally derived from acetyl and malonyl‐CoA through a polyketide synthase–mediated biosynthetic pathway in many fungi belonging to the Basidiomycota and Ascomycota divisions (Li et al. 2011; Calcott et al. 2018; Hafez Ghoran et al. 2022). Among these, genera such as *Aspergillus*, *Fusarium*, *Penicillium*, *Talaromyces*, *Thermomyces*, *Arthrinium* and *Alternaria* are considered the most important producers of a wide diversity of quinones and their derivatives (Christiansen et al. 2021; Yang et al. 2020). The genus *Thermomyces* is phylogenetically closely related to common mesophilic fungi *Aspergillus* spp. and *Penicillium* spp. but contains a largely reduced genome and displays a concise metabolic profile (Chu et al. 2010; Guo et al. 2012; Yang et al. 2023; Chen et al. 2023; Li, He, et al. 2024; Li, Wang, et al. 2024). Importantly, *Thermomyces* is a predominant group of thermophilic fungi capable of growing and thriving at high temperatures of 45°C–60°C and displays two distinct pigment patterns at optimum growth temperatures of 45°C–55°C and a minimum growth temperature of 37°C (Maheshwari et al. 2000; Chen et al. 2023; Yang et al. 2023; Li, He, et al. 2024; Li, Wang, et al. 2024). Our previous studies have revealed that *Thermomyces dupontii* produces much darker colonies at a relatively low growth temperature of 37°C, attributed to the accumulation of carviolin A, a predominant anthraquinone metabolite that is widely distributed in fungi and plants (Li, Wang, et al. 2024; Masi and Evidente 2020). The mutant lacking anthraquinone biosynthesis (Δ*An*) exhibited a loss of both dark pigmentation and ergosterol‐mediated endocytosis. Instead, the mutant Δ*An* produced an unprecedented oxygen‐free ergosterene, resulting in self‐isolation and minimal interactions with its surroundings. Interestingly, the primary anthraquinone in the fungus, carviolin A, was shown to reduce ferric to ferrous ions and transport the latter across the fungal membrane (Li, Wang, et al. 2024). Eventually, we found that the thermophilic fungus can utilize the anthraquinones to induce and regulate a powerful extracellular Fenton reaction, which can not only degrade the organic matter in the surrounding environment but also increase the ambient temperature (Li, Wang, et al. 2024; Wu, Wang, et al. 2025; Dai et al. 2025). Furthermore, we observed that the ATP content of WT with extracellular Fenton reactions was significantly lower than that of mutant Δ*An* without extracellular Fenton reactions (Wu, Wang, et al. 2025; Dai et al. 2025; Wu, Zhou, et al. 2025). It seems that anthraquinones mediate a trade‐off relationship between extracellular Fenton reactions and intracellular ATP levels.

This study is a continuation of our work on the natural functions of pigments in thermophilic fungal adaptation to environmental temperature changes (Yang et al. 2023; Chen et al. 2023; Li, He, et al. 2024; Li, Wang, et al. 2024). In this study, we focused on the thermophilic fungus *T. dupontii* , whose survival under low‐temperature stress (e.g., 37°C) has been linked to extracellular Fenton chemistry, a process driven by anthraquinones to degrade organic matter for energy production (Li, Wang, et al. 2024). Using the drug affinity responsive target stability (DARTS) method, which exploits the reduced protease susceptibility of the target protein upon drug binding (Pai et al. 2015; Ren et al. 2021), we identified potential target proteins for carviolin A. Additionally, transcriptomic analysis was performed on both wild type (WT) and the mutant Δ*An*. These analyses highlighted the involvement of a hal protein in the anthraquinone‐based metabolic response to cold stress. In vitro enzyme catalysis experiments revealed that the hal enzyme is responsible for the chlorination of carviolin A.

To further investigate pigment composition in the fungus, we employed ultra‐high performance liquid chromatography (UHPLC) coupled with ultraviolet/visible (UV/VIS) detection and high‐resolution accurate‐mass (HRAM) quadrupole electrospray ionisation (ESI) mass spectrometry. These chemical analyses confirmed the presence of two major chlorinated anthraquinones, each with distinct skeletons and colours. Bioinformatic analysis revealed that the *hal* gene is located in the *An* gene cluster for the carviolin A biosynthesis. Through mutation and metabolic analysis, combined with detailed chemical investigation, we found that the *hal* gene also contributed to the chlorination of dihydroanthraquinones and dichlorination of anthraquinones. Detailed chemical investigation revealed that the *hal* gene is involved in introducing chlorine into carviolin A and emodin, generating novel monochlorinated and dichlorinated anthraquinones with distinct pigmentation and structural diversity. Notably, disruption of the chlorination pathways significantly reduced the ATP levels in the mutant by impairing electron production. These results suggested that *T. dupontii* utilises the chlorination of anthraquinones to elevate ATP formation for fungal vitality under cold stress, compensating for the decrease in ATP content caused by the extracellular Fenton reaction mediated by anthraquinones.

## 2 Results

### 2.1 Hal Is a Potential Target of Carviolin A

**FIGURE 1:**
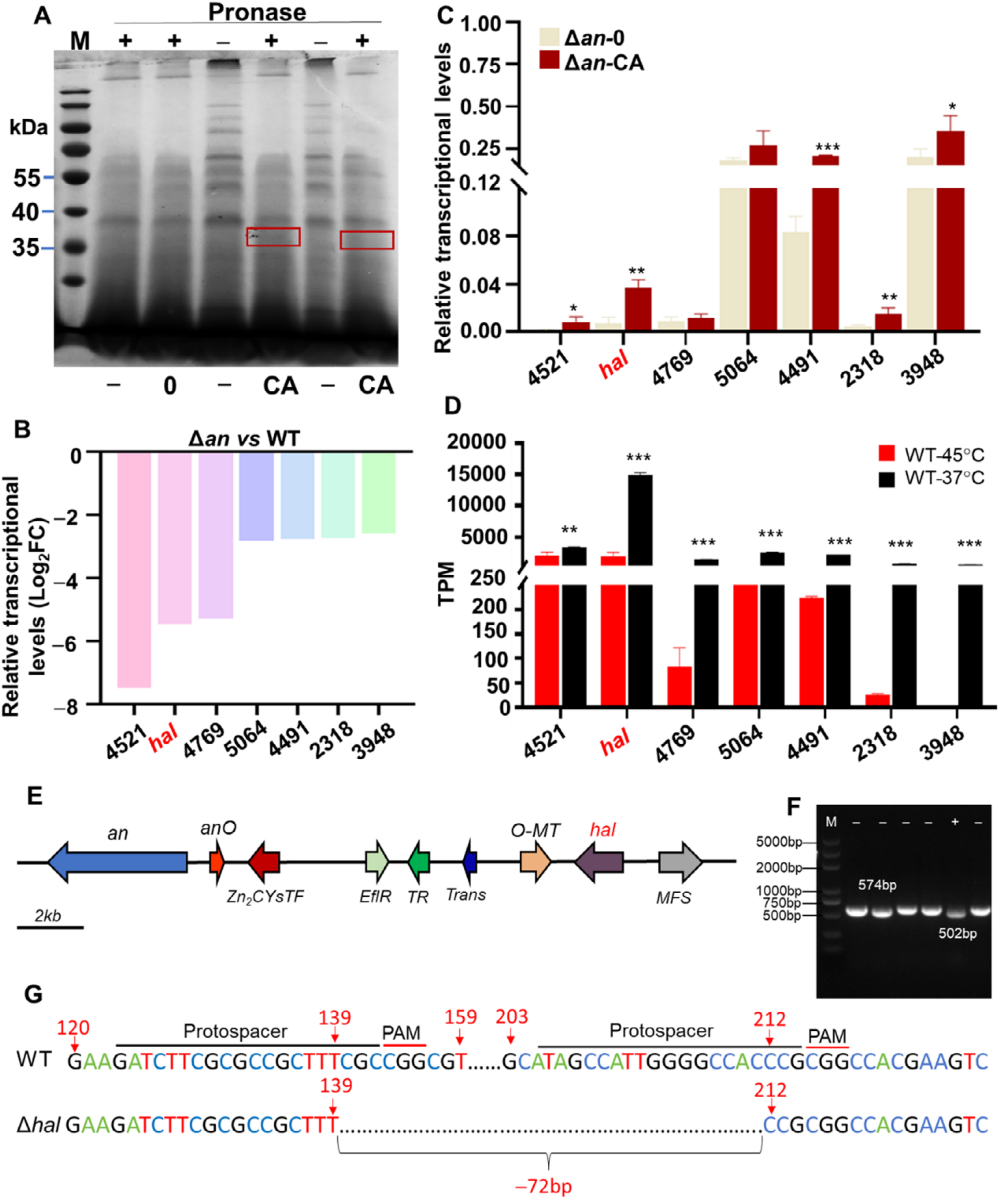
Characterisation of Hal as the potential target of the anthraquinones in thermophilic fungus. (A) DARTS analysis of the protected protein band (in red box) which displayed the decreased protease susceptibility upon anthraquinone binding. (B) Transcriptomic analysis of Δ*An* and WT exhibit the significantly down‐regulated transcriptional levels of seven genes, which encode the potential protein targets. (C) RT‐PCR analysis displayed that carviolin A increased the transcriptional levels of five potential target genes in Δ*An*. 0: Solvent only; CA: 20 μM carviolin A. (D) Transcriptomic analysis of WT at 37°C and 45°C exhibited that seven potential gene targets displayed significantly increased transcriptional levels at 37°C vs. at 45°C. (E) Gene *hal* located in the *an* gene cluster for the biosynthesis of anthraquinones. (F) PCR analysis of transformants with disruption of gene *hal*. (G) Sequencing analysis of the mutant Δ*hal*. *p < 0.05; **p < 0.01; ***p < 0.001.

The total protein in *T. dupontii* at 37°C was extracted and incubated with carviolin A, followed by the addition of the proteolytic enzyme pronase. A comparison of the bands on the SDS‐PAGE gel between the carviolin A treatment and the control revealed protected bands appearing at 37 KDa (Figure 1A). Mass spectrometry analysis identified a total of 151 protein sequences (Table S1). To refine the potential targets, we performed the transcriptomic analysis on both WT and the mutant Δ*An*. Among the top 30 down‐regulated genes in Δ*An* versus WT, seven genes coincided with the potential protein targets identified in the DARTS analysis (Figure 1B). These seven genes include the NADH‐cytochrome B5 reductase gene (*4521*), a flavin‐dependent halogenase gene (*hal*) (*7038*), an NAD(P)H‐binding protein gene (*4769*), an aldehyde dehydrogenase gene (*5064*), a tyrosinase gene (*4491*), a prenyltransferase gene (*2318*) and an NAD(P)H quinone dehydrogenase gene (*3948*). Notably, the gene *hal* was located within the *An* gene cluster, which contained three genes responsible for carviolin A biosynthesis: a polyketide synthase gene (*An*), an anthrone oxygenase gene (*AnO*) and an O‐methyltransferase (*O‐MT*). The An enzyme is responsible for producing the first key precursor, emodin anthrone, which is subsequently oxidised and O‐methylated to yield carviolin A (**1**).

To evaluate the effect of carviolin A on transcriptional levels of these seven target genes, the mutant Δ*An* was chemically complemented with carviolin A, while the solvent DMSO served as the control group. RT‐PCR analysis revealed that five genes, including *hal*, exhibited significantly increased transcriptional levels in the carviolin A‐treated group compared to the control (Figure 1C). Further transcriptional analysis of WT at 37°C and 45°C demonstrated that all seven genes, including *hal*, were significantly up‐regulated in WT at 37°C compared to 45°C. Notably, *hal* was the most up‐regulated gene, not only among these seven but across all genes analysed in WT under the same conditions (Figure 1D,E). This finding suggested that *hal* plays a critical role in the fungal response to cold stress.

### 2.2 Hal Is Responsible for the Chlorination of Anthraquinones

We used a modified CRISPR‐Cas9 technology suitable for thermophilic fungi to disrupt the *hal* gene in *T. dupontii* (Li, He, et al. 2024; Li, Wang, et al. 2024). A mutant strain, Δ*hal*, with a 140–211 bp deletion in the first exon of the gene *hal*, was generated and confirmed through sequencing analysis (Figure 1E−G). Metabolic analysis of extracts from the mutant Δ*hal* and WT at 37°C and 45°C revealed that the primary anthraquinone, carviolin A (**1**) was present in both strains at both temperatures. A substantial accumulation of carviolin A (**1**) was observed at 37°C compared to 45°C, consistent with the previous findings that cold stress induced significant accumulation of carviolin A (**1**) in *T. dupontii* .

**FIGURE 2:**
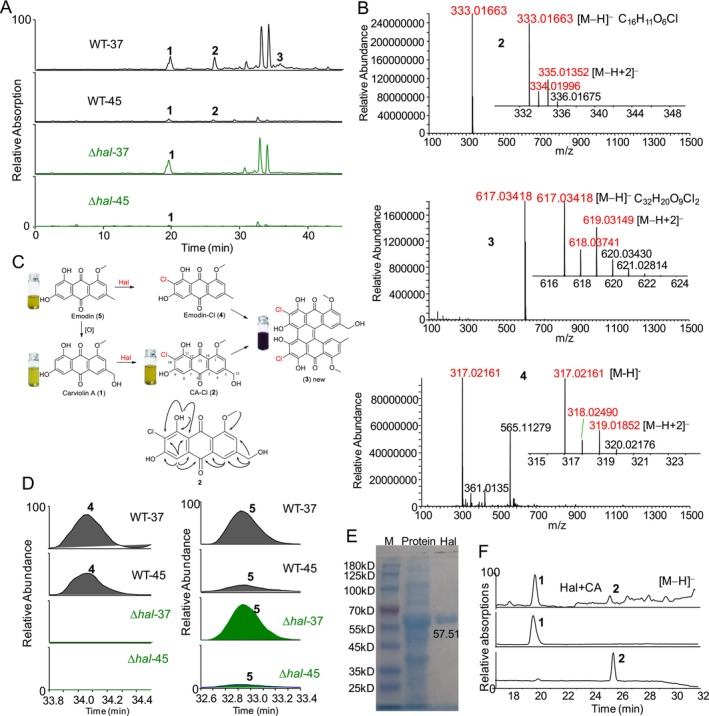
Characterisation of the role of hal in the chlorination of anthraquinones in thermophilic fungus. (A) HPLC‐MS analysis of Δ*hal* and WT at 37°C and 45°C exhibited the major target metabolite peaks (**2** and **3**) related to gene *hal* and its precursor (**1**). (B) Mass spectra of the major target metabolites (**2** and **3**) and trace target metabolite **4**. (C) The biosynthesis of chlorinated anthraquinones (**2**–**4**) derived from their precursors (**1** and **5**) in thermophilic fungus and the key HMBC of metabolite **2**. (D) HPLC‐MS analysis of Δ*hal* and WT at 37°C and 45°C exhibited the trace target metabolite peak (**4**) related to gene *hal* and its precursor (**5**). (E) Purified Hal protein from heterologously expressed *E. coli* BL21*‐hal*. (F) HPLC‐MS analysis displayed the Hal‐mediated chlorination of carviolin A (**1**) to **2** via in vitro reaction.

Notably, disruption of the gene *hal* resulted in the absence of two major metabolite peaks in Δ*hal* compared to WT, regardless of temperature (Figure 2A). Importantly, the target metabolites **2** and **3** exhibited the [M − H]^−^ peaks at *m/z* 333 and 617, respectively, in their negative ESI spectra (Figure 2B), accompanied by corresponding strong [M − H + 2]^−^ peaks at *m/z* 335 and 619, indicating the presence of chlorine in both metabolites. Furthermore, the molecular formula of C_16_H_11_O_6_Cl for metabolite **2** indicates that it contains one more chlorine but one less hydrogen than carviolin A (**1**). An ethyl acetate (EtOAc) layer from the 50 L fermentation filtrate of the *T. dupontii* WT strain at 37°C. The EtOAc layer was evaporated to dryness and then subjected to repeated chromatography using macroporous resin, Sephadex LH‐20 gel, silica gel and RP‐18, yielding the two major target metabolites **2** and **3**. Interestingly, metabolite **2** is yellow, while metabolite **3** is purple. Their structures were elucidated through comprehensive NMR and high‐resolution MS analysis (Figures S1–S12 and Table S2).

The ^1^H NMR and ^13^C NMR spectra (Figures S1 and S2) of metabolite **2** were quite similar to those of carviolin A (**1**), except that metabolite **2** had one additional olefinic quaternary carbon and one fewer olefinic methine compared to carviolin A (**1**) (Figures S13 and S14). The HSQC spectrum of metabolite **2** enabled the assignment of all protons bonded to carbons. The ^1^H − ^1^H coupling relationships and ^1^H − ^13^C long‐range correlations in the 2D NMR spectra of **2** (Figures S3–S6) led to the identification of the extra chlorine attached to C‐10, specifically 10‐chlorine carviolin A (**2**, CA‐Cl). This is the first report of the ^3^C NMR, and DEPT spectra and 2D NMR of metabolite **2** (Figures S2–S6). The ^1^H NMR and ^13^C NMR spectra of metabolite **3** (Figures S7 and S8) indicated that **3** is a chlorinated anthraquinone dimer (Figure 2C), consistent with the molecular formula of C_32_H_20_O_9_Cl_2_ for metabolite **3**, as determined from the HRESIMS spectra. Analysis of the HSQC, COSY and HMBC spectra of metabolite **3** (Figures S9–S12) revealed that metabolite **3** is a dimer of **2** fused with another known chlorinated anthraquinone, namely, 10‐chlorine analogue (emodin‐Cl, **4**) derived from emodin (**5**) (Figure 2C).

The only difference between emodin‐Cl (**4**) and CA‐Cl (**2**) is that metabolite **2** has one additional oxygen at C‐15, a distinction confirmed by the HRESI MS spectrum of emodin‐Cl (**4**), which exhibited a distinct [M − H + 2]^−^ ion peak at *m/z* 319.01852 accompanying the [M − H]^−^ ion peak at *m/z* 317.02161 (Figure 2C). Detailed analysis of metabolic profiles of WT and the mutant Δ*hal* at 37°C and 45°C revealed that emodin‐Cl (**4**) was also present in WT but absent in Δ*hal*, regardless of temperature (Figure 2D). This finding confirms that the unique *hal* gene in the *An* cluster is responsible for the chlorination of anthraquinones, such as carviolin A (**1**) and emodin (**5**).

The *hal* gene was cloned from *T. dupontii* and heterologously expressed in *Escherichia coli* BL21. The successfully expressed mutant strain underwent large‐scale fermentation, and the target protein, hal, was isolated and purified (Figure 2E). The catalytic activity of the hal protein on the chlorination of the major anthraquinone, carviolin A, was then assessed in vitro. HPLC‐PDA/MS analysis of the ethyl acetate extract of the reaction mixture revealed a target peak for metabolite **2** (Figure 2F), confirming that the hal protein is the target responsible for the chlorination of anthraquinones.

### 2.3 Chlorination Contributed to Fungal Pigmentation and Cell Integrity of *T. dupontii*

**FIGURE 3:**
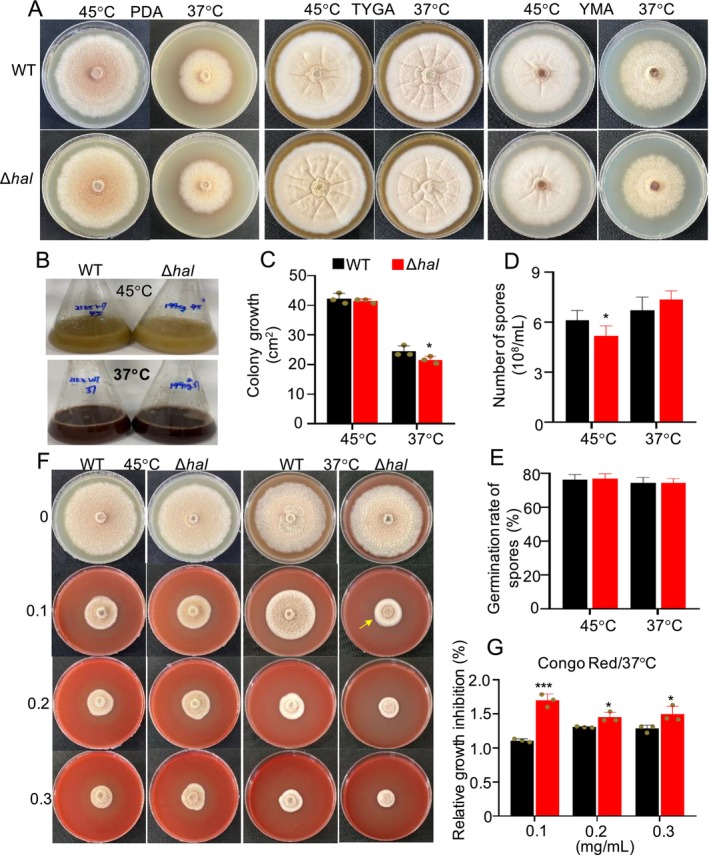
Evaluation of the function of chlorination in pigmentation, phenotype and resistance of thermophilic fungus at different temperatures. (*n* = 3) (A) Pictures of the mutant Δ*hal* and WT on three different media, PDA, TYGA and YMA at 37°C and 45°C. (B) Pictures of the mutant Δ*hal* and WT cultivated in PDB in shaking flasks at 37°C and 45°C. (C) Comparison of colony growth the mutant Δ*hal* (red) and WT (black). (D) Comparison of spore formation between the mutant Δ*hal* (red) and WT (black). (E) Comparison of spore germination rates between the mutant Δ*hal* (red) and WT (black). (F) Pictures of colony growth of the mutant Δ*hal* and WT on PDA with Congo Red. (G) Comparison of Congo Red resistance between the mutant Δ*hal* (red) and WT (black). Relative growth inhibition rate (%) = [(Colony area in blank culture medium—Colony area under stress conditions)/Colony area in blank culture medium] × 100%. *p < 0.05; **p < 0.01; ***p < 0.001.

Phenotypic analysis revealed that the Δ*hal* mutant exhibited a yellowish colour on three different media compared to WT, regardless of temperature (Figure 3A). Under shaking conditions at 45°C, the Δ*hal* culture was lighter in colour than WT (Figure 3B). These results indicated the lack of anthraquinone chlorination led to changes in fungal pigmentation. Although colony growth on PDA was quite similar between the Δ*hal* mutant and WT at both temperatures (Figure 3C), the Δ*hal* mutant at 45°C displayed significantly reduced spore formation compared to WT (Figure 3D). No significant difference in spore germination rates was observed between the Δ*hal* mutant and WT (Figure 3E). Congo Red, a water‐soluble diazo dye, is commonly used to induce cell wall integrity stress or assess fungal cell wall mutants (Lima et al. 2021; Liu et al. 2021). As expected, the Δ*hal* mutant showed significantly reduced colony growth on PDA with 0.1–0.3 μM Congo Red compared to WT (Figure 3F,G), suggesting that chlorination enhances fungal resistance to Congo Red by strengthening the cell wall.

### 2.4 Hal Is Involved in Chlorination of Dihydroanthraquinone

**FIGURE 4:**
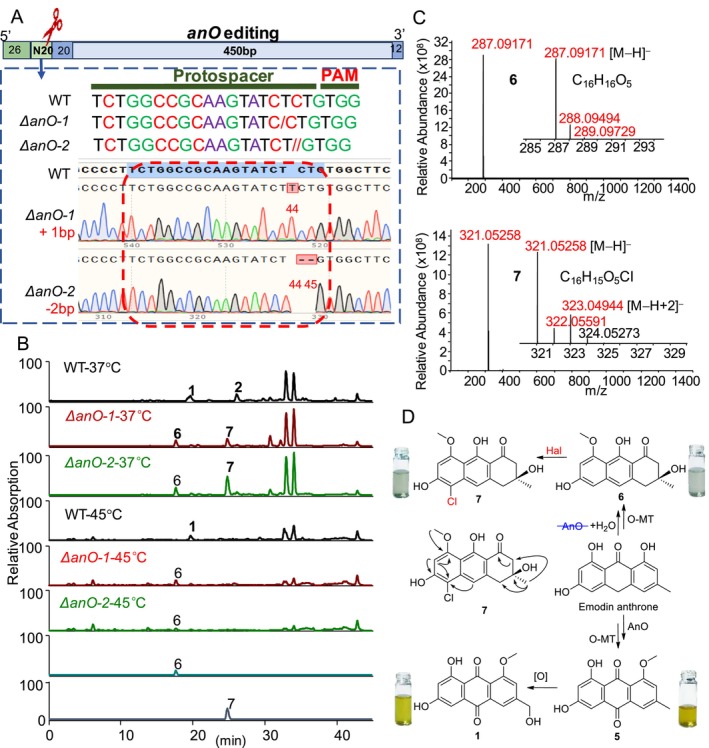
Broad chlorination capacity of Hal in yielding new chlorinated anthraquinone in the mutant Δ*anO* under low temperature. (A) Schematic diagram of the gene editing and sequencing analysis of the mutant Δ*anO*. (B) HPLC‐MS analysis of Δ*anO* and WT at 37°C and 45°C exhibited that disruption of the gene *anO* resulted in the presence of **6** and **7** in Δ*anO* whereas the major target metabolite **7** is produced only at 37°C. (C) Mass spectra of the major target metabolites **6** and its chlorinated derivative **7**. Metabolite **7** displayed a diagnostic ion peak [M − H + 2]^−^ in the MS spectrum and **6** does not. (D) The biosynthetic pathway and the key HMBC relations for chlorinated anthraquinone **7** that is produced by the reduction and subsequent chlorination of the common precursor emodin anthrone, which can be also oxidised to **5** and then to **1** in thermophilic fungal WT.

Previous work has shown that the *An* gene is involved in the production of the key precursor, emodin anthrone, which is further oxidised by AnO and O‐methylated by O‐MT to yield carviolin A (**1**). Based on the structures of the known chlorinated anthraquinones in fungi and plants, it is reasonable to infer that the anthraquinone chlorination reaction occurs on carviolin A (**1**) and its demethyl and dehydroxyl analogues. Interestingly, chlorinated derivatives of deoxygenated anthraquinones have been rarely reported. To further investigate this, we applied a modified CRISPR‐Cas9 method to disrupt the *AnO* gene in *T. dupontii* . Two Δ*AnO* mutants were generated: one with an extra base pair at position 44 and the other with two missing base pairs at positions 44–45, and both mutants were confirmed by sequencing analysis (Figure 4A). Metabolic analysis of the extracts from the Δ*AnO* mutants and WT at 37°C and 45°C revealed that the main anthraquinone, carviolin A (**1**), was present in WT at both temperatures, along with its chlorinated analogue, CA‐Cl (**2**), in WT at 37°C (Figure 4B). Notably, both carviolin A (**1**) and CA‐Cl (**2**) were absent in the two Δ*AnO* mutants at both temperatures. Instead, two additional metabolite peaks (**6** and **7**) were observed in the two Δ*AnO* mutants at 37°C (Figure 4B). Among these, metabolite peak **7** was strongly accumulated at 37°C compared to 45°C (Figure 4B). The target metabolites **6** and **7** exhibited [M − H]^−^ peaks at *m/z* 287.09171 and 321.05258, respectively, in their negative ESI spectra (Figure 4C), corresponding to molecular formulas of C_16_H_16_O_5_ for **6** and C_16_H_15_O_5_Cl for **7**, suggesting that **7** had one more chlorine but one less hydrogen than metabolite **6**. Furthermore, a strong [M − H + 2]^−^ peak at *m/z* 323.04944 accompanying the [M − H]^−^ peak at *m/z* 321.05258 in the negative ESI spectrum of **7** confirmed the presence of chlorine in metabolite **7** (Figure 4C).

An EtOAc layer of the 40 L fermentation filtrate of the mutant Δ*AnO‐2*, cultivated at 37°C, the EtOAc layer was evaporated to dryness and then purified by repeated column chromatography on macroporous resin, silica gel, Sephadex LH‐20 gel and RP‐18. This process yielded two major target metabolites **6** and **7**. The structures of these metabolites were elucidated based on extensive NMR and HRMS analysis. Metabolite **6** was identified as a known dihydroanthraquinone (Figure 4D) based on matching 1D and 2D NMR data (Figures S15–S20) with previously reported spectra. The ^1^H NMR and ^13^C NMR spectra (Figures S21 and S22) of metabolite **7** were highly similar to those of metabolite **6**, with the exception of an additional olefinic quaternary carbon and one fewer olefinic methine in **7**. The HSQC spectrum of **7** allowed for the assignment of all protons−carbon bonds (Figure S23). The ^1^H‐^1^H coupling relationship and ^1^H‐^13^C long‐range correlations (Figures S24–S26) in the 2D NMR spectra of **7** revealed the presence of an additional chlorine atom attached to C‐8, identifying it as the 8‐chlorine analogue of **6** (Figure 4D). The experimental ECD spectrum of **7** (Figure S27) was consistent with the calculated ECD spectrum for **7**, with an S‐configuration at C‐3, suggesting that the structure of **7** as described in Figure 4D. Therefore, metabolite **7** was characterised as a novel chlorinated dihydroanthraquinone. These findings suggest that the *hal* gene is involved in the chlorination of both anthraquinones and dihydroanthraquinones, highlighting the versatility of hal‐mediated chlorination in substrate selection.

### 2.5 Hal Has the Capacity of Double Chlorination of Anthraquinone

**FIGURE 5:**
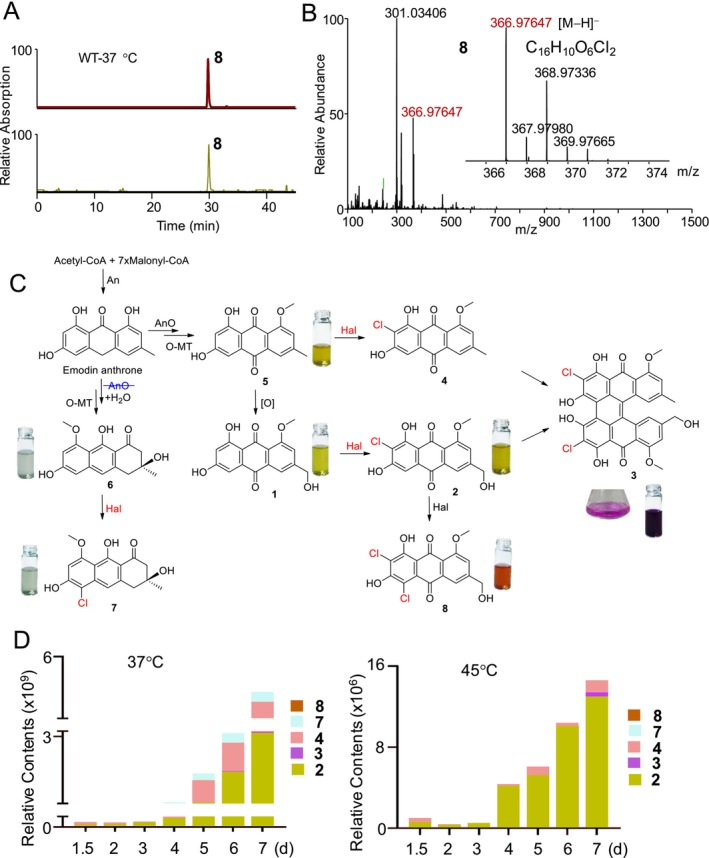
(A) Metabolic analysis of dichlorinated anthraquinone **8** in WT at 37°C. (B) Mass spectra of dichlorinated anthraquinone **8**. (C) The biosynthetic pathways for all the chlorinated anthraquinones from WT and the mutants. (D) Comparison of the contents of all the chlorinated anthraquinones in WT at 37°C and 45°C revealed that the chlorinated anthraquinones were accumulated in the late stage and the total contents of the chlorinated anthraquinones in WT at 37°C are 100 times than those at 45°C.

In artificial chemical synthesis, dichlorination of alkenes remains a prevalent method for incorporating chlorine atoms into organic molecules. Naturally occurring dichlorinated anthraquinones, however, are rarely reported in fungi and plants. This raises the question of whether the *hal* gene could be involved in the dichlorination reaction in *T. dupontii* . Since carvoilin A (**1**) and its monochlorinated metabolite **2** were the predominant anthraquinones in *T. dupontii* , we searched for the dichlorinated metabolites of carvoilin A based on molecular weight in the metabolic profiles of *T. dupontii*. Interestingly, a target trace peak corresponding to metabolite **8** appeared in the metabolic profiles of *T. dupontii* (Figure 5A), showing a [M − H]^−^ peak at *m/z* 366.97647, along with a strong [M − H + 2]^−^ peak at *m/z* 368.97336, in the negative ESI spectrum (Figure 5B). A molecular formula of C_16_H_10_O_6_Cl_2_ was assigned to metabolite **8**, indicating that **8** contained one more chlorine and one less hydrogen than **2**. To isolate the trace target metabolite **8**, an EtOAc layer of the 60 L fermentation filtrate of the *T. dupontii* WT strain, cultivated at 37°C, was carefully examined. Eventually, metabolite **8** was purified, and its structures were elucidated as a new dichlorinated derivative of carvoilin A based on extensive NMR and HRMS analysis. The ^1^H NMR and ^13^C NMR spectra (Figures S28 and S29) of metabolite **8** were highly similar to those of **2**, with the exception that **8** had one additional olefinic quaternary carbon and one fewer olefinic methine. The HSQC spectrum of **8** enabled the assignment of all proton−carbon bonds (Figure S30). The ^1^H‐^1^H coupling relationship and ^1^H‐^13^C long‐range correlations (Figures S31–S33) in the 2D NMR spectra of **8** revealed the presence of chloride atoms at C‐8 and C‐10 (Figure 5C). Interestingly, the dichlorinated metabolite **8** is dark red, whereas the monochlorinated **2** is yellow (Figure 5C).

The possible biosynthetic pathways for all the chlorinated metabolites from the *T. dupontii* WT and its mutants were also proposed (Figure 5C). A comparison of the chlorinated metabolites in WT at 37°C and 45°C revealed that the total content of the chlorinated metabolites at 37°C was more than 100 times higher than that at 45°C. Notably, chlorinated metabolites accumulated in the late stage of fungal growth at both temperatures (Figure 5D).

### 2.6 *T. dupontii* Utilises Chloride Ion via Chlorination for ATP Formation

**FIGURE 6:**
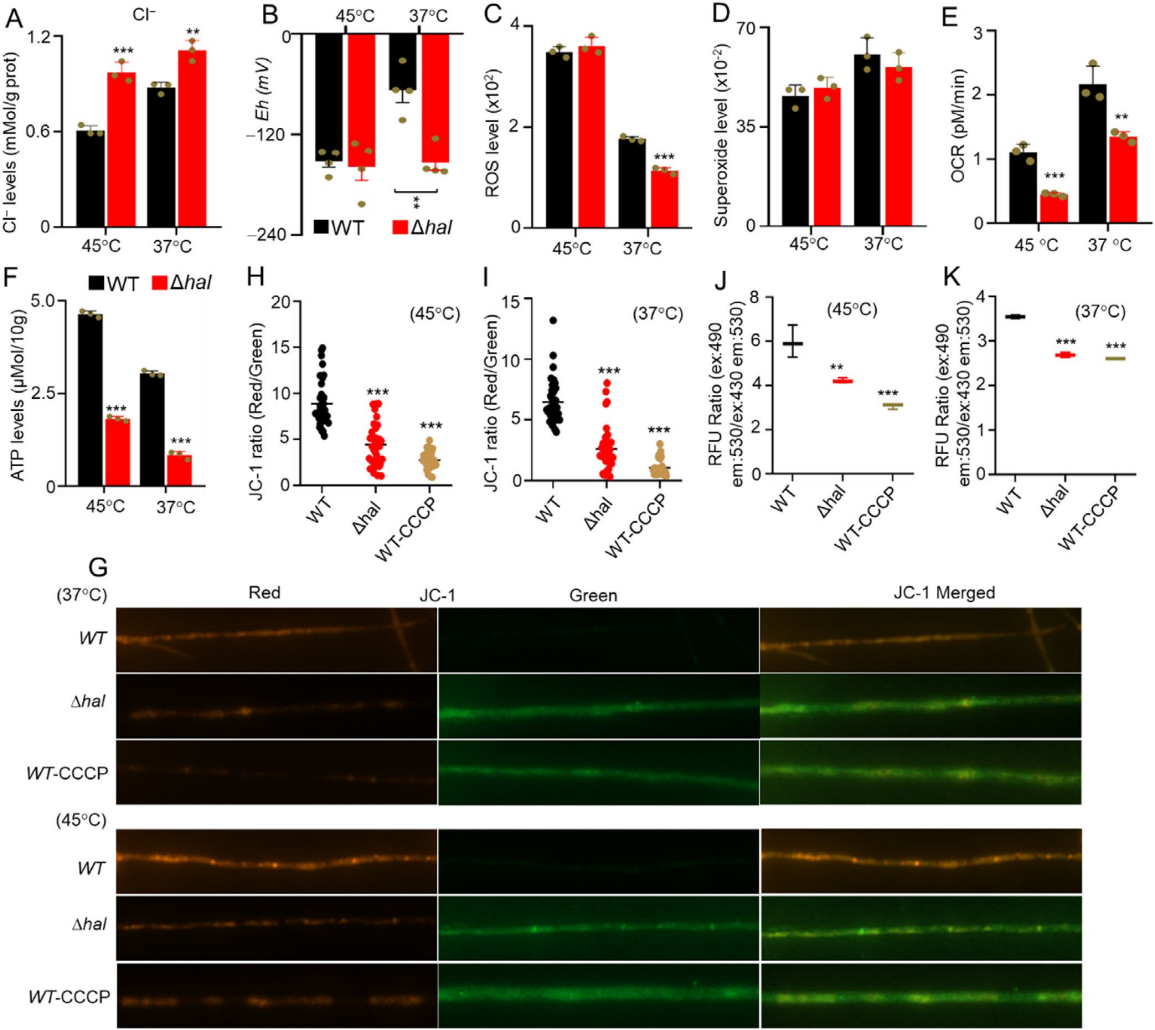
Evaluation of the role of chlorination in controlling Cl^−^ and redox homeostasis, and energy production in thermophilic fungus at 37°C and 45°C. (*n* ≥ 3) (A) Comparison of the Cl^−^ levels the mutant Δ*hal* (red) and WT (black). (B) Comparison of redox potential between the mutant Δ*hal* (red) and WT (black). (C, D) Analysis of ROS (C) and superoxide (D) in the mutant Δ*hal* (red) and WT (black). (E, F) Comparison of OCR (E) and ATP levels (F) between the mutant Δ*hal* (red) and WT (black) at 37°C and 45°C. (G–I) Comparison of the mitochondrial membrane potential between the mutant Δ*hal* and WT at 37°C and 45°C. (J, K) Comparison of the proton levels between the mutant Δ*hal* and WT at 37°C and 45°C. *p < 0.05; **p < 0.01; ***p < 0.001.

The chemical mechanism of chlorination involved chlorine replacing one hydrogen in anthraquinone, with electrons from the chloride ion being donated. A bioassay to evaluate chloride ion levels in the Δ*hal* mutant and WT was conducted. The Δ*hal* mutant showed significantly higher chloride ion levels than WT (Figure 6A). Redox potential analysis revealed no significant change in the redox potential of the mutant Δ*hal* between 37°C and 45°C, while WT exhibited an increase from −152.0 mV at 45°C to −67.5 mV at 37°C (Figure 6B), suggesting that the chlorination reaction contributed to fungal redox homeostasis in responding to low temperature. Further analysis showed that the levels of reactive oxygen species (ROS) were significantly lower in the Δ*hal* mutant than in WT at 37°C (Figure 6C), while no significant change in superoxide levels was observed between the Δ*hal* mutant and WT at both temperatures (Figure 6D). A previous study suggested that flavin‐dependent halogenases (FDHs) are classified as electrophilic halogenases, which use flavin and oxygen to mediate chlorination (Agarwal et al. 2017; Andorfer and Lewis 2018; Phintha et al. 2020). A bioassay measuring oxygen consumption rates (OCR) revealed that the Δ*hal* mutant exhibited significantly lower OCR than WT at both temperatures (Figure 6E).

Since redox reactions are intrinsically linked to energy metabolism, ATP levels were analysed in the Δ*hal* mutant and WT at 37°C and at 45°C. Notably, the Δ*hal* mutant showed significantly lower ATP levels than WT at both temperatures (Figure 6F). In particular, low temperature exacerbated the reduction in ATP levels in Δ*hal* relative to WT. Further analysis revealed that the mitochondrial membrane potentials in the Δ*hal* mutant were strongly decreased compared to those in WT at both temperatures. The reduction was almost equivalent to that caused by the positive control, carbonylcyanide‐3‐chlorophenylhydrazone (CCCP) (Figure 6G−I), a protonophore that uncouples the proton gradient in the inner mitochondrial membrane, thereby inhibiting ATP synthesis (Demine et al. 2019; Zhang et al. 2021). Given that ATP synthesis is driven by proton gradients, the proton levels were evaluated in the Δ*hal* mutant and WT at both temperatures. Interestingly, the Δ*hal* mutants exhibited a substantial decrease in proton levels compared to WT, similar to the positive control CCCP (Figure 6J,K). These results indicated that chlorination played a crucial role in ATP formation by enhancing mitochondrial membrane potential.

### 2.7 Chlorination Is Essential for Electron Production in *T. dupontii*

**FIGURE 7:**
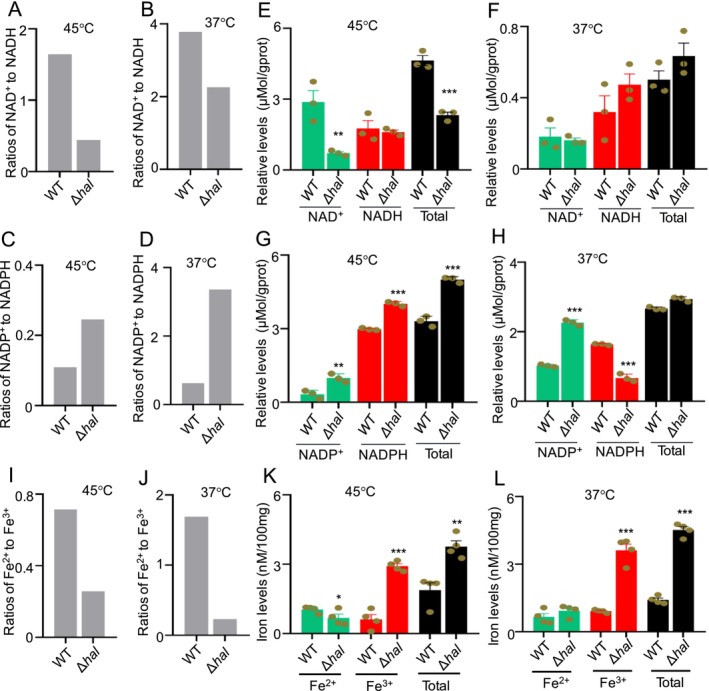
Evaluation of the role of chlorination in controlling electron production in thermophilic fungus at 37°C and 45°C. (*n* ≥ 3) (A–D) Comparison of the ratios of NAD^+^/NADH and NADP^+^/NADPH in the mutant Δ*hal* and WT at 37°C and at 45°C. (E, F) Comparison of the contents of NAD^+^, NADH and total levels, in the mutant Δ*hal* and WT at 37°C and at 45°C. (G, H) Comparison of the contents of NADP^+^, NADPH and total levels in the mutant Δ*hal* and WT at 37°C and at 45°C. (I, J) Comparison of the ratios of Fe^2+^/Fe^3+^ in the mutant Δ*hal* and WT at 37°C and at 45°C. (K, L) Comparison of Fe^2+^, Fe^3+^ and total levels, in the mutant Δ*hal* and WT at 37°C and at 45°C. For (A–D and I, J): Data represent normalised averages of three replicates, as these are derived ratios (rather than direct measurements) without error bars. *p < 0.05; **p < 0.01; ***p < 0.001.

In the electron transport chain, ATP is produced by removing electrons from NADH, a reduced form of nicotinamide adenine dinucleotide (NAD^+^). The addition of a phosphate group from ATP to NAD^+^ leads to nicotinamide adenine dinucleotide phosphate (NADP^+^) (Xiao et al. 2018; Schiuma et al. 2024). The main difference between NAD and NADP is that NAD^+^/NADH is used in cellular respiration for ATP formation, whereas NADP^+^/NADPH is mainly used in the anabolic reactions. Normally, NADPH is the most abundant form of NADP inside the cell, which acts as a reducing agent. Interestingly, the Δ*hal* mutant exhibited significantly decreased NAD^+^/NADH ratios (Figure 7A,B) but significantly increased NADP^+^/NADPH ratios (Figure 7C,D), compared with WT at both temperatures. At 45°C, the Δ*hal* mutant exhibited a significant decrease in total NAD content but a marked increase in total NADP content, compared to WT (Figure 7E−H). These results indicated that the chlorination reaction controls the total contents of NAD and NADP during temperature fluctuations. At low temperature of 37°C, NADH level was significantly increased in the Δ*hal* mutant vs. WT (Figure 7E−H), which was consistent with a significantly decreased ATP level in the Δ*hal* mutant at 37°C. Meanwhile, NADPH level was significantly decreased in the Δ*hal* mutant vs. WT (Figure 7E−H), suggesting that the Δ*hal* mutant was in the oxygenated state and lack of electron in the Δ*hal* mutant.

Iron is a crucial metal electron carrier in redox reactions within cells. Recent studies have demonstrated that anthraquinones are involved in iron reduction and transport in *T. dupontii* (Li, Wang, et al. 2024). Therefore, the contents and composition of free iron ions in the Δ*hal* mutant and WT were analyzed at 37°C and 45°C. Notably, compared to WT, the Fe^2+^/Fe^3+^ratio in the Δ*hal* mutant was significantly reduced at both temperatures, primarily due to the marked increase in Fe^3+^ levels (Figure 7I−L). The elevated Fe^3+^ levels in the mutant Δ*hal* also resulted in a substantial increase in the total free iron ion levels at both temperatures (Figure 7K,L). The reduced Fe^2+^/Fe^3+^ ratio in the Δ*hal* mutant is consistent with the lack of electron donation by chloride ions. Additionally, the increased Fe^3+^ levels are in line with the decreased mitochondrial membrane potentials.

## 3 Discussion

To date, halogen moieties are present in many pharmaceutical and agrochemical products, as well as other valuable materials, and are widely used across various sectors of the chemical industry as artificially synthetic intermediates and products (Cantillo and Kappe 2017; Chiodi and Ishihara 2023; Jeschke 2025). The introduction of a halogen atom can profoundly affect the bioactivity and physicochemical properties of small molecules (Agarwal et al. 2017; Latham et al. 2018; Wang, He, et al. 2024). This effect has been exploited in agrochemical and medicinal chemistry, with a large proportion of drugs in clinical trials or on the market containing halogen atoms (Smith et al. 2014; Wang et al. 2014; Fang et al. 2019). Most studies mainly focused on the chemical mechanisms of hal in the halogenation of reactive compounds, which can significantly improve the properties of organic molecules, enabling them to effectively and selectively regulate biological targets in vivo.

This study investigates the chemical and genetic foundation of a pigment system that has evolved across fungi, contributing to the vivid hues that ornament their phenotype and enable survival under limited nutritional conditions. Our biochemical and transcriptomics analysis revealed that the hal enzyme serves as a target protein for anthraquinones in thermophilic fungus under cold stress. Through a combination of genetic manipulation, metabolic analysis and chemical investigation, we characterised the role of the hal enzyme in anthraquinone‐driven colour shifts. Furthermore, we demonstrated a strong correlation between anthraquinones' chlorination and the late stage of fungal growth. Given that the spectrum of hues observed in many fungi is often attributed to the selective deposition of anthraquinones and dihydroanthraquinones in their phenotypes, it is highly plausible that hal, or other enzymes with halogenase activity, play a role in these colour variations across a wide range of fungal species.

Phenotypic analysis revealed that the colour transition in the fungal colony during the late stage appears to be a direct result of changes in anthraquinone metabolism, mediated by hal enzyme, which leads to the production of chemically distinct pigments. This is consistent with the accumulation of chlorinated anthraquinones, as indicated by our chemical analysis of pigment composition. Although multiple genetic factors likely influence the overall colour phenotype of the fungus, our results demonstrate that substantial colour shifts in anthraquinones pigmentation can be achieved through subtle element substitution in the chemical structure. This simplicity may explain why evolutionary transitions from greenish‐yellow/yellow to red and purple are so common in fungi and plants.

The ability of organisms to harness environmental elements to generate energy for growth during temperature fluctuation is a critical survival strategy. The adaptive significance of fungal colour variations remains poorly understood, despite numerous studies reporting that cold‐adapted fungi in the cryosphere produce a variety of pigments as a protective strategy against ecological stresses such as low temperature, oxidative stresses and ultraviolet radiation (Rafiq et al. 2019; Sajjad et al. 2020; Garcia‐Lopez et al. 2021). These pigments make fungi a potential source for natural pigment production.

In this study, we found that the *hal* gene plays a critical role in vital cellular functions. Our mutation and functional analysis demonstrate the *hal* gene is deeply involved in the ATP formation through electron transfer to iron. Our findings provide an example of a biosynthetic gene being co‐opted for a new function, exerting its effects during the late stage by modifying available compounds, thereby minimizing potential pleiotropic consequences and energy consumption. Although most of the Earth's biosphere is exposed to temperatures as low as 5°C year‐round, which was once considered too harsh for life, long‐term cold adaptation leads to an increase in both the number and activity of mitochondria to support rising ATP consumption (Guderley 2004; Talbot et al. 2004). This temperature range is even more hostile for thermophilic fungi, which struggle to grow at temperatures below 37°C. Our results showed that the *hal* gene deficiency led to a 90% reduction in ATP content in *T. dupontii* at 37°C and a 60% reduction at 45°C, indicating that the *hal* gene plays a decisive role in ATP formation in the thermophilic fungus at relatively low temperatures.

Interestingly, the significant decrease in ATP content did not lead to reduced growth in the Δ*hal* mutant under relatively low temperature. Previous studies have reported that the basal metabolism maintains homeostatic control of high NAD^+^/NADH and low NADP^+^/NADPH redox ratios in resting cells (Chen et al. 2024; Scherschel et al. 2024). Conversely, in proliferating cells, basal catabolic metabolism shifted towards anabolism, resulting in low NAD^+^/NADH and high NADP^+^/NADPH redox ratios to support biomass synthesis and cell growth (Da Veiga Moreira et al. 2015). Thus, the low NAD^+^/NADH and high NADP^+^/NADPH ratios induced by the lack of *hal* might contribute to the fungal growth and proliferation of the Δ*hal* mutant. This data suggested that the *hal* gene might be involved in inhibiting proliferating cells during the late stage of the thermophilic fungal growth by controlling the NAD^+^/NADH and NADP^+^/NADPH redox ratios. Our previous study indicated that *T. dupontii* WT at 37°C can perform extracellular Fenton chemistry for the fungal growth (Li, Wang, et al. 2024). The data from this study suggested that the Δ*hal* mutant can also perform the extracellular Fenton chemistry for fungal growth, although its ATP production capacity is greatly reduced due to lack of *hal* gene (Li, Wang, et al. 2024; Dai et al. 2025; Wang et al. 2025; Wu, Wang, et al. 2025). The high NADP^+^/NADPH and low NAD^+^/NADH in the Δ*hal* mutant indicated that NAD^+^/NADH is involved in ATP production, whereas NADP^+^/NADPH is responsible for extracellular Fenton chemistry.

In summary, we found that a flavin‐dependent halogenase gene *hal*, responsible for the anthraquinone chlorination, can enhance fungal pigmentation and promote ATP synthesis that underpins fungal colour and vitality under low‐temperature stress, elucidating the ecological significance of chlorinated anthraquinones in fungal adaptation. This study uncovers the nature function of the chlorination reaction in energy production and opens avenues for exploring the adaptive significance of colour traits more broadly, providing critical insights into natural product diversification and biotechnological applications.

## 4 Materials and Methods

### 4.1 Organisms, Plasmids and Culture Conditions

The fungus *T. dupontii* 2155 ( *T. dupontii* ) and corresponding mutants were stored in the Microbial Library of the Germplasm Bank of wild species from Southwest China (Kunming, China). *T. dupontii* 2155 and mutants Δ*hal* were cultured on potato dextrose agar (PDA, potato (Kunming, China)) 200 g L^−1^, glucose (Solarbio, Beijing, China) 10 g L^−1^, agar ((Solarbio, Beijing, China) 15 g L^−1^). All the bioassays were conducted in 9 cm diameter Petri dishes. *T. dupontii* were cultured on PDA plates at 45°C for 7 days or 37°C for 14 days. PDA and TYGA medium (10 g/L tryptone (Oxoid, Basingstoke, UK), 10 g/L glucose, 5 g/L yeast extract (Oxoid, Basingstoke, UK), 5 g/L molasses(Acmec, Shanghai, China), 18 g/L agar) and YMA medium (5 g/L yeast extract (Oxoid, Basingstoke, UK), 5 g/L Maltose (Sangon Biotech, Shanghai, China), 18 g/L agar) and YG medium (5 g/L yeast extract, 20 g/L glucose) were used to determine the growth and other phenotypic traits of the fungus *T. dupontii* WT and mutants Δ*hal*. *Escherichia coli* strain DH5α (Tsingke, Beijing, China) was used to construct and store recombinational plasmids. *E. coli* BL21(DE3) (Tsingke, Beijing, China) was used for protein expression. (Yang et al. 2023; Li, He, et al. 2024; Li, Wang, et al. 2024). All assays were repeated three times (*n* = 3).

### 4.2 Fungal Strains and Protein Extraction

*T. dupontii* was cultured on PDA at 45°C for 7 days. Three to four mycelial disks (5–6 mm diameter) were inoculated into 500 mL flasks containing 250 mL PDB. Cultures were incubated at 37°C on a rotary shaker (180 rpm) for 7 days. Mycelia were harvested by filtration through four layers of sterile lens paper (10 × 15 cm, NEWSTAR Industry, Hangzhou, China) in a funnel, washed with 10 mL of phosphate‐buffered saline (PBS, pH 7.5), and resuspended in PBS supplemented with phosphatase inhibitors. Mycelia were disrupted by ultrasonication on ice for 30 min, using a pulse sequence of 6 s on and 6 s off. The lysate was centrifuged at 15,000 × g for 10 min at 4°C to collect the supernatant, and the protein concentration was quantified using a BCA assay kit (Beyotime, China) (Chen et al. 2019).

### 4.3 Binding Assay With CA

Total protein supernatant (200 μg per 50 μL aliquot) was treated with carviolin A (CA) at final concentrations of 10 μM and 50 μM, with DMSO as a control. The mixtures were incubated at 21°C with shaking at 300 rpm for 2 h. After incubation, 1 μL of protease solution (0.5 μg/mL) was added, followed by hydrolysis at 37°C for 5 min. Reactions were terminated by addition of 10 μL of 6× SDS‐PAGE loading buffer, followed by boiling for 10 min. Differential protein bands were separated by SDS‐PAGE. CA‐bound proteins, which are protected from protease‐mediated degradation due to conformational stability, were excised from the gel and identified by via liquid chromatography–tandem mass spectrometry (LC–MS/MS) based on specific peptide sequences (Pai et al. 2015; Ren et al. 2021).

### 4.4 Screening and Identification of CA Target Proteins

To validate and refine the candidate proteins identified with DARTS, transcriptome data were integrated to select seven proteins that were significantly downregulated in the Δ*An* knockout strain at 37°C. To investigate whether these proteins are regulated by or interact with CA, the Δ*An* strain was cultured in fermentation medium supplemented with 20 μM CA (DMSO as control). Following 7 days of incubation, fungal hyphae were harvested for RNA extraction and subsequent analysis via reverse transcription‐polymerase chain reaction (RT‐PCR) (Pai et al. 2015; Ren et al. 2021).

### 4.5 Mutant Construction via Thermophilic CRISPR/Cas9 System

One SpCas9 expression plasmid, designated as p‐TrpC‐NLS‐coSpCas9‐NLS, This plasmid contains a hygromycin resistance gene (HygR) as the selection marker, and a codon‐optimised SpCas9 gene (coSpCas9) fused with nuclear localization signal (NLS) sequences. The expression of coSpCas9 is driven by the fungal constitutive promoter TrpC. (Huang et al. 2020). A sgRNA expression plasmid was also utilised, which contains a tRNA‐Gly self‐processing system driven by the native promoter ptRNAGly from *T. dupontii* . The protospacer and corresponding PAM sequences for each sgRNA were selected from the 5′ region of the putative catalytic domain in each target gene, and are listed in Table S3 of the Supporting Information. Each sgRNA sequence served as one half of the primer, which contained a 20 bp overlap with the sgRNA expressing plasmid p‐tRNA‐Gly‐sgRNA. This plasmid harbours a single tRNA^Gly system driven by the promoter p*tRNA*^Gly, which is functional in *T. dupontii* . The sgRNA expression plasmids for targeted gene editing in *T. dupontii* were constructed using PCR and In‐Fusion cloning methods. Subsequently, both the SpCas9 expression plasmid p‐TrpC‐NLS‐co SpCas9‐NLS and the sgRNA expression plasmids were transformed into *T. dupontii* protoplasts following the protocol described in a previous study. Transformat colonies were selected following incubation at 45°C for 2–4 days, and each single colony was transferred to a new plate containing 200 μg/mL hygromycin B. Following 5 days of incubation at 45°C, genomic DNA was extracted from putative transformants and subjected to PCR verification to confirm the genomic integration of the target genes. All mutants with target gene deficiencies were screened and further validated by PCR. (Yang et al. 2023; Li, He, et al. 2024; Li, Wang, et al. 2024).

### 4.6 Fungal Morphology, Conidial Production and Germination

1. Mycelial plugs (9 mm diameter) from 7‐day‐old WT and mutant colonies were inoculated onto PDA, TYGA and YMA media (5 replicates/strain). Cultures were incubated at 45°C and 37°C to observe growth rate and colony morphology. Colony diameters were measured after 7 days (45°C) and 10 days (37°C) to calculate colonisation area for fungal growth. Relative growth inhibition rate (%) = [(Colony area in blank culture medium—Colony area under stress conditions)/Colony area in blank culture medium] × 100%.
2. For sporulation assessment, WT and mutant strains were cultured on PDA at 45°C for 8 days and 37°C for 13 days. Hyphae and spores were harvested with 5 mL of sterile water, filtered through four layers of lens paper, and centrifuged (12,000 r/min, 2 min, 4°C). The spore pellet was washed with 1 mL of sterile water to prepare spore suspensions, which were counted using a haemocytometer (1/400 mm^2^, 25 × 16) with the formula: Spore number = (spores in small cells/*n*) × 400 × 10^4^ × dilution factor. Spore germination was evaluated by inoculating 10 μL of 1 × 10^8^/mL spore suspension into 1 mL YG medium at 45°C and 37°C (180 r/min). Germination rate (germinated spores/total spores) was recorded every 2 h (Li, He, et al. 2024).

### 4.7 Stress Tolerance Bioassays

WT and mutant colonies, initiated from 9 mm hyphal discs, were incubated on PDA plates (control) or PDA supplemented with Congo red (0.1, 0.2, 0.3 mg/mL) at 45°C for 7 days or 37°C for 13 days, respectively. These assays evaluated cell wall perturbation and acidification levels (Chen et al. 2022).

### 4.8 Metabolic Analysis

*T. dupontii* strains were cultured on PDA at 45°C for 7 days. Mycelial disks (5–6 mm, 3–4 pieces) were inoculated into 500 mL flasks containing 250 mL PDB and incubated at 37°C or 45°C for 7 days with shaking (180 rpm). The fermentation broth was extracted with 250 mL of ethyl acetate at a ratio of 1:1 (v/v), followed by ultrasonic treatment for 40 min with shaking once every 10 min. The organic layer was concentrated to dryness under reduced pressure, resuspended in 1 mL methanol, and 200 μL of this solution was further diluted in 800 μL chromatographic‐grade methanol, filtered through a 0.22 μm membrane, and analysed by HPLC‐MS/MS. HPLC‐MS/MS analysis was performed on a Q Extractive Focus system (Thermofisher) with a PDA detector and Orbitrap mass detector, using a CAPCELL PAK C18 column (Shiseido, 5 μm, 4.6 mm × 250 mm) and electrospray ionisation (positive/negative modes). Mobile phases were 0.1% formic acid in water (A) and 0.1% formic acid in acetonitrile (B) at a flow rate of 1 mL/min. The gradient program was: 0–2 min (10% B), 10 min (25% B), 30 min (50% B), 35 min (90% B), 36–40 min (95% B), 40–45 min (10% B). Column temperature was 40°C, injection volume 10 μL and UV spectra were recorded at 196–400 nm. Data were processed using Compound Discoverer 3.0 software (Thermofisher) (Chen et al. 2023; Yang et al. 2023; Li, He, et al. 2024; Wang, Song, and Jiao 2024).

### 4.9 Halogenase Cloning, Expression and Purification

Cloning: Total RNA was extracted from *T. dupontii* 2155 using the Tiangen RNA Purification Kit. cDNA was synthesised with the SuperScript First‐Strand Synthesis System (Takara) using oligo‐dT primers. The intron‐free *hal* gene (GME199_g) was amplified from cDNA using primers hal‐For/hal‐Rev, inserted into the NcoI/XhoI‐digested pET28a plasmid via infusion cloning, and confirmed by sequencing. The resulting recombinant pET28a‐*hal* plasmid was transformed into *E. coli* BL21(DE3) for heterologous protein expression.

Expression and purification: A 12.5 mL overnight culture of *E. coli* BL21 harbouring the *hal* expression construct was inoculated into 250 mL LB medium (supplemented with 50 μg/mL kanamycin) in 500 mL flasks. When OD₆₀₀ reached 0.4–0.6, expression was induced with 0.2 mM IPTG (16°C, overnight). Cells were centrifuged (8000 × g, 10 min, 4°C), resuspended in 20 mL buffer (100 mM sodium phosphate pH 7, 10 mM imidazole, 150 mM NaCl), and lysed by ultrasonication (2 s on/3 s off, 30 min, 150 W, 4°C). Lysates were centrifuged (12,000 × g, 10 min, 4°C), and supernatants were incubated with Ni‐NTA agarose (4°C, 2 h). The resin was washed with 20 mL buffer (50 mM sodium phosphate pH 7, 80 mM imidazole, 150 mM NaCl), and Hal was eluted with 5 mL buffer (100 mM sodium phosphate pH 7, 500 mM imidazole, 50 mM NaCl). The protein eluate was transferred to an ultrafiltration centrifugal device with a 30 kDa molecular weight cut‐off (MWCO) and centrifuged at 3000 × g until the retentate volume was reduced to approximately 200 μL. Subsequently, 1 mL of exchange buffer (50 mM sodium phosphate, pH 7.0, containing 10% (v/v) glycerol) was added to the retentate, followed by centrifugation at 3000 × g to reduce the volume to ~200 μL again. This dilution‐concentration cycle was repeated twice more, resulting in a total of three buffer exchanges. After the final concentration step yielded ~200 μL of retentate, the concentrated sample was carefully recovered. The retentate was then aliquoted into 50 μL portions per tube and stored at −80°C. Purified Hal was analysed by SDS‐PAGE and quantified using a BCA kit (Chen et al. 2019; Bai et al. 2020; Ni et al. 2024).

### 4.10 Hal‐Catalysed Halogenations

In vitro reactions (200 μL) were conducted in HEPES buffer (pH 7.5) containing 10 mM NaCl, 20 mM glucose, 10 μM flavin adenine dinucleotide disodium salt hydrate (FAD) (HY‐B1654, MedChemExpress, USA), 10 μM Nicotinamide Adenine Dinucleotide (NAD) (606–68‐8, Rhawn, Shanghai, China), 35 U/mL catalase from the bovine liver (Cat. No. C8070, Solarbio, Beijing, China), 9 U/mL glucose dehydrogenase from *Bacillus* (GDH) (Lot #H1520116, Aladdin, Shanghai, China), 2.5 μM flavin reductase (Fre) (HY‐P71709, MedChemExpress, USA), 50 μM Hal protein and 1 mM carviolin A (substrate). Reactions were initiated by the addition of substrate, followed by incubation at 28°C with shaking (600 rpm) for 12 h. Reactions were quenched with equal volume of MeOH, centrifuged at 15,000 rpm for 10 min, and the resulting supernatants analysed by HPLC‐MS (Mondal et al. 2021; Jiang et al. 2022; Peh et al. 2023).

### 4.11 Chlorine Level Assay

The Chlorine Assay Kit (E‐BC‐K189‐M) was used for Chlorine leve analysis. Briefly, 100 mg mycelia were washed with cold ddH₂O, followed by lysis via ultrasonication in ddH₂O (2 s on/3 s off, 30 min, 150 W, 4°C). The resulting mixture was centrifuged (12,000 rpm, 10 min, 4°C), 10 μL supernatant was collected. For extracellular levels, 1 mL filtrate was centrifuged (12,000 rpm, 10 min, 4°C) and 10 μL supernatant was used. All Samples were mixed with 250 μL assay buffer, incubated in the dark for 5 min, and then measured at OD 460 nm.

### 4.12 Redox Potentials

One hundred milligram of mycelia was harvested and washed three times with cold PBS. The washed mycelia were then resuspended in 30 mL of M9 buffer, and redox potential measurements were performed using a redox potential detector (Wang, Yan, et al. 2024).

### 4.13 ROS/Superoxide Assay

The reactive oxygen species (ROS) and superoxide were measured using the ROS/Superoxide Detection Assay Kit (#ab139476, Abcam) following the manufacturer's instructions. Briefly, 20 mg of mycelia were harvested in a 2 mL centrifuge tube and were treated with 500 μL of ROS/Superoxide Detection Solution and incubated for 60 min at 37°C or 45°C in the dark. The stained mycelia were analyzed by a microplate reader. Oxidative Stress Detection Reagent (Green, Ex/Em 490/525 nm) was used for the evaluation of total ROS, and Superoxide Detection Reagent (Orange, Ex/Em 550/620 nm) for superoxide (Yang et al. 2023; Li, Wang, et al. 2024).

### 4.14 Oxygen Consumption Rate Test

The extracellular oxygen consumption rate (OCR) was measured using the Extracellular Oxygen Consumption Assay Kit (#ab197243, Abcam, UK) following the manufacturer's instructions. Briefly, approximately 10 mg of fungal hyphae were transferred into a 1.5 mL centrifuge tube containing 1 mL of PDB medium and mixed using a vortex oscillator. Subsequently, 100 μL of the mixture (equivalent to 1 mg hyphae per well) was inoculated into a 96‐well microplate. Each well was then supplemented with 8 μL of reconstituted extracellular O₂ consumption reagent and 100 μL of prewarmed high‐sensitivity mineral oil (37°C). The plates were incubated at 37°C and monitored kinetically using the CLARIOstar energy metabolism monitor over a 4‐h period (read every 2 min, Ex/Em = 380/650 nm). Basal OCR levels were calculated based on the slope of the kinetic curve (Li, Wang, et al. 2024).

### 4.15 ATP Assay

The ATP levels in fungal mycelia were directly measured using the ATP Detection Assay Kit (S0026, Beyotime, Shanghai, China). Briefly, 100 mg of mycelia were harvested and washed three times with cold phosphate‐buffered saline (PBS). The washed mycelia were resuspended in 300 μL of ATP lysis buffer and homogenised on ice using a homogeniser. The homogenate was then centrifuged at 12,000 rpm for 10 min at 4°C, and 20 μL of the supernatant was collected for analysis. Subsequently, 100 μL of ATP assay buffer was added to the supernatant, mixed thoroughly and after 2 s, luminescence was measured using a microplate reader set to luminometer mode.15. Mitochondrial membrane potential assay.

### 4.16 Mitochondrial Membrane Potential Assay

The mitochondrial membrane potential was directly measured using the 3,3′‐Diethyloxacarbocyanine (DiOC_2_(3) (abs42008034, Absin, Shanghai, China)). Briefly, sterilised cover slips were inserted at a 45° angle into PDA medium and fungal colonies (9 mm in diameter) were inoculated onto the medium. The cultures were incubated at 45°C for 5 days or at 37°C for 7 days, allowing the aerial hyphae to grow onto and cover the cover slips. To assess mitochondrial membrane potential, the cover slips with aerial hyphae were incubated with DiOC_2_(3) staining solution. One aliquot of the staining solution was supplemented with the uncoupling agent carbonyl cyanide 3‐chlorophenylhydrazone (CCCP) as a positive control. The samples were incubated at 37°C for 30 min, washed three times with PBS, and immediately observed under a fluorescence microscope (ZEISS, Germany). Fluorescence excitation was performed at 484 nm, and emission wavelengths of 530 nm (green fluorescence) and 610 nm (red fluorescence) were recorded. The red‐to‐green fluorescence ratios were calculated from 30 randomly selected visual fields for analysis (Xia et al. 2024).

### 4.17 Proton Levels Assay

The proton dynamics were directly measured using the 2′,7′‐Bis‐(2‐carboxyethyl)‐5‐(and‐6)‐carboxyfluorescein, acetoxymethyl ester (BCECF‐AM) (40701ES50, Yeasen, Shanghai, China). Briefly, spores were collected and washed three times with Hank's Balanced Salt Solution (HBSS). Germination was induced in YG medium for 4 h, after which BCECF‐AM was added to a final concentration of 25 μM. A parallel sample was supplemented with the uncoupling agent carbonyl cyanide 3‐chlorophenylhydrazone (CCCP) as a positive control. The samples were incubated at 37°C for 30 min, washed three times with HBSS, and the fluorescence ratio was measured. Excitation wavelengths of 490 nm and 430 nm were used, with emission recorded at 530 nm (Xia et al. 2024).

### 4.18 NAD ^+^/NADH Assay

NAD^+^/NADH levels were measured using an NAD^+^/NADH Assay Kit (E‐BC‐K804‐M, Elabscience, Wuhan, China) according to the manufacturer's protocol. Briefly, 100 mg of fungal mycelia were harvested and washed three times with cold PBS. The mycelia were then resuspended in NAD^+^/NADH assay buffer and homogenized on ice. The homogenate was centrifuged at 12,000 rpm for 10 min at 4°C, and 20 μL of the supernatant was used for total NAD^+^/NADH detection. For NADH‐specific detection, 100 μL of the supernatant was heated at 60°C for 30 min to degrade NAD^+^, followed by centrifugation at 12,000 rpm for 5 min at 4°C. Then, 20 μL of the resulting supernatant was used for analysis. The reaction mixture was prepared by adding 120 μL of Working Solution and 40 μL of color‐developing reagent, which was incubated for 30 min in the dark. Absorbance was measured immediately at 450 nm using a microplate reader.

### 4.19 NADP ^+^/NADPH Assay

NADP^+^/NADPH levels were measured using an NADP^+^/NADPH Assay Kit (E‐BC‐K803‐M Elabscience, Wuhan, China) according to the manufacturer's instructions. Briefly, 100 mg fungal mycelia were harvested, washed thrice with cold PBS, resuspended in NADP^+^/NADPH assay buffer, and homogenised on ice. The homogenate was centrifuged at 12,000 rpm for 10 min at 4°C. For total NADP^+^/NADPH detection, 50 μL supernatant was used. For NADPH‐specific detection, 100 μL supernatant was heated at 60°C for 30 min to degrade NADP^+^, recentrifuged at 12,000 rpm for 5 min at 4°C, and 50 μL of the resulting supernatant was analysed. The reaction was initiated with 10 μL colour‐developing reagent, incubated 5 min in the dark, and absorbance measured immediately at 450 nm using a microplate reader. (Pecchillo Cimmino et al. 2022).

### 4.20 Iron Measurement

Iron levels (Fe^2+^ and Fe^3+^) were measured using an Iron Assay Kit (#I291, Dojindo, Japan). Briefly, 100 mg fungal mycelia were harvested and washed three times with cold PBS. The washed mycelia were resuspended in iron assay buffer and homogenised on ice using a homogeniser. The homogenate was centrifuged at 12,000 rpm for 10 min at 4°C, and 100 μL of the supernatant was collected for analysis. Iron assay buffer and a reducing agent were added to the supernatant, mixed thoroughly, and incubated following the manufacturer's instructions. Subsequently, the solution was incubated with the iron probe for 1 h in the dark. Absorbance was measured immediately at 593 nm using a microplate reader (Li, Wang, et al. 2024; Wang et al. 2025).

## Author Contributions

**Donglou Wang:** investigation, writing – original draft, validation, methodology, visualization, formal analysis, data curation. **Gang Dai:** methodology, validation, investigation, formal analysis, data curation, visualization. **Jiangbo He:** investigation, methodology, validation, data curation, formal analysis. **Huiwen Si:** formal analysis, data curation, investigation, validation, methodology. **Chunhua Liao:** data curation, validation. **Wenjie Wang:** validation, data curation. **Zhangxin Zuo:** validation, data curation. **Shuhong Li:** validation, data curation. **Xuemei Niu:** conceptualization, investigation, writing – original draft, writing – review and editing, visualization, validation, project administration, supervision, resources, data curation, funding acquisition, methodology.

## Conflicts of Interest

The authors declare no conflicts of interest.

## Supporting information


**Table S2:** The ^1^H and ^13^C NMR data of compounds **1**–**3** and **6**–**8**.
**Table S3:** Amplification of sgRNA and validation primers.
**Figure S1:** The ^1^H NMR of metabolite **2** (27–334).
**Figure S2:** The ^13^C NMR of metabolite **2** (27–334).
**Figure S3:** The HSQC of metabolite **2** (27–334).
**Figure S4:** The COSY of metabolite **2** (27–334).
**Figure S5:** The HMBC of metabolite **2** (27–334).
**Figure S6:** The NOESY of metabolite **2** (27–334).
**Figure S7:** The ^1^H NMR of metabolite **3** (purple).
**Figure S8:** The ^13^C NMR of metabolite **3** (purple).
**Figure S9:** The HSQC spectrum of metabolite **3** (purple).
**Figure S10:** The COSY spectrum of metabolite **3** (purple).
**Figure S11:** The HMBC spectrum of metabolite **3** (purple).
**Figure S12:** The NOESY spectrum of metabolite **3** (purple).
**Figure S13:** The ^1^H NMR of CA (**1**).
**Figure S14:** The ^13^C NMR of CA (**1**).
**Figure S15:** The ^1^H NMR of metabolite **6** (27–288).
**Figure S16:** The ^13^C NMR of metabolite **6** (27–288).
**Figure S17:** The HSQC of metabolite **6** (27–288).
**Figure S18:** The COSY of metabolite **6** (27–288).
**Figure S19:** The HMBC of metabolite **6** (27–288).
**Figure S20:** The NOESY of metabolite **6** (27–288).
**Figure S21:** The ^1^H NMR of metabolite **7** (27–321).
**Figure S22:** The ^13^C NMR of metabolite **7** (27–321).
**Figure S23:** The HSQC of metabolite **7** (27–321).
**Figure S24:** The COSY of metabolite **7** (27–321).
**Figure S25:** The HMBC of metabolite **7** (27–321).
**Figure S26:** The NOESY of metabolite **7** (27–321).
**Figure S27:** The ECD spectrum of metabolite **7**.
**Figure S28:** The ^1^H NMR spectrum of metabolite **8** (367).
**Figure S29:** The ^13^C NMR spectrum of metabolite 8 (367).
**Figure S30:** The HSQC spectrum of metabolite **8** (367).
**Figure S31:** The COSY spectrum of metabolite **8** (367).
**Figure S32:** The HMBC spectrum of metabolite **8** (367).
**Figure S33:** The NOESY spectrum of metabolite **8** (367).
**Figure S34:** (A) PCR analysis of gene *hal*. (B) Isolation and purification of the protein Hal from *hal‐*containing *E. coli* BL 21. (C–D) HPLC‐PDA/MS analysis displayed the chlorination of carviolin A (1) via Hal in vitro.

**Table S1:** mbt270254‐sup‐0002‐TableS1.xlsx.

## Data Availability

The data that supports the findings of this study are available in the Supporting Information of this article.
