# Supplementary material for: Novel Anthraquinone Chlorination Contributes to Pigmentation and ATP Formation in Thermomyces dupontii
Source: Microb Biotechnol. 2025 Oct 22;18(10):e70254. doi: 10.1111/1751-7915.70254 (PMC12541888; doi:10.1111/1751-7915.70254)
Supplement: Supplementary file 1 — Table S2: The 1H and 13C NMR data of compounds 1–3 and 6–8. Table S3: Amplification of sgRNA and validation primers. Figure S1: The 1H NMR of metabolite 2 (27–334). Figure S2: The 13C NMR of metabolite 2 (27–334). Figure S3: The HSQC of metabolite 2 (27–334). Figure S4: The COSY of metabolite 2 (27–334). Figure S5: The HMBC of metabolite 2 (27–334). Figure S6: The NOESY of metabolite 2 (27–334). Figure S7: The 1H NMR of metabolite 3 (purple). Figure S8: The 13C NMR of metabolite 3 (purple). Figure S9: The HSQC spectrum of metabolite 3 (purple). Figure S10: The COSY spectrum of metabolite 3 (purple). Figure S11: The HMBC spectrum of metabolite 3 (purple). Figure S12: The NOESY spectrum of metabolite 3 (purple). Figure S13: The 1H NMR of CA (1). Figure S14: The 13C NMR of CA (1). Figure S15: The 1H NMR of metabolite 6 (27–288). Figure S16: The 13C NMR of metabolite 6 (27–288). Figure S17: The HSQC of metabolite 6 (27–288). Figure S18: The COSY of metabolite 6 (27–288). Figure S19: The HMBC of metabolite 6 (27–288). Figure S20: The NOESY of metabolite 6 (27–288). Figure S21: The 1H NMR of metabolite 7 (27–321). Figure S22: The 13C NMR of metabolite 7 (27–321). Figure S23: The HSQC of metabolite 7 (27–321). Figure S24: The COSY of metabolite 7 (27–321). Figure S25: The HMBC of metabolite 7 (27–321). Figure S26: The NOESY of metabolite 7 (27–321). Figure S27: The ECD spectrum of metabolite 7. Figure S28: The 1H NMR spectrum of metabolite 8 (367). Figure S29: The 13C NMR spectrum of metabolite 8 (367). Figure S30: The HSQC spectrum of metabolite 8 (367). Figure S31: The COSY spectrum of metabolite 8 (367). Figure S32: The HMBC spectrum of metabolite 8 (367). Figure S33: The NOESY spectrum of metabolite 8 (367). Figure S34: (A) PCR analysis of gene hal. (B) Isolation and purification of the protein Hal from hal‐containing E. coli BL 21. (C–D) HPLC‐PDA/MS analysis displayed the chlorination of carviolin A (1) via Hal in vitro. [file MBT2-18-e70254-s002.docx]

SUPPORTING INFORMATION

**Novel Anthraquinone Chlorination Contributes to Pigmentation and ATP Formation in *Thermomyces dupontii***

Donglou Wang,^1,⊥^ Gang Dai,^1,⊥^ Jiangbo He,^2,⊥^ Huiwen Si,^1^ Chunhua Liao,^1^ Wenjie Wang,^1^ Zhangxin Zuo,^1^ Shuhong Li,^1^ Xuemei Niu^1,*^

1 State Key Laboratory for Conservation and Utilization of Bio-Resources in Yunnan &Yunnan key Laboratory of Basic Research and Innovative Application for Green Biological Production, Key Laboratory for Microbial Resources of the Ministry of Education, School of Life Sciences, Yunnan University, Kunming 650091, P. R. China.

2 Kunming Key Laboratory of Respiratory Disease, Kunming University, Kunming 650214, P. R. China

Running head: Anthraquinone Chlorinations-Mediated Pigmentation and ATP Formation

^⊥^These authors contributed equally.

*Corresponding author: [xmniu@ynu.edu.cn](mailto:niuxm@yahoo.com)

**Supplementary Tables**

**Table S2.** The ^1^H and ^13^C NMR data of compounds **1**−**3** and **6**−**8**.

**Table S3.** Amplification of sgRNA and validation primers.

**Supplementary Figures**

**Figure S1.** The ^1^H NMR of metabolite **2** (27-334).

**Figure S2.** The ^13^C NMR of metabolite **2** (27-334).

**Figure S3.** The HSQC of metabolite **2** (27-334).

**Figure S4.** The COSY of metabolite **2** (27-334).

**Figure S5.** The HMBC of metabolite **2** (27-334).

**Figure S6.** The NOESY of metabolite **2** (27-334).

**Figure S7.** The ^1^H NMR of metabolite **3** (purple).

**Figure S8.** The ^13^C NMR of metabolite **3** (purple).

**Figure S9.** The HSQC spectrum of metabolite **3** (purple).

**Figure S10.** The COSY spectrum of metabolite **3** (purple).

**Figure S11.** The HMBC spectrum of metabolite **3** (purple).

**Figure S12.** The NOESY spectrum of metabolite **3** (purple).

**Figure S13.** The ^1^H NMR of CA (**1**).

**Figure S14.** The ^13^C NMR of CA (**1**).

**Figure S15.** The ^1^H NMR of metabolite **6** (27-288).

**Figure S16.** The ^13^C NMR of metabolite **6** (27-288).

**Figure S17.** The HSQC of metabolite **6** (27-288).

**Figure S18.** The COSY of metabolite **6** (27-288).

**Figure S19.** The HMBC of metabolite **6** (27-288).

**Figure S20.** The NOESY of metabolite **6** (27-288).

**Figure S21.** The ^1^H NMR of metabolite **7** (27-321).

**Figure S22.** The ^13^C NMR of metabolite **7** (27-321).

**Figure S23.** The HSQC of metabolite **7** (27-321).

**Figure S24.** The COSY of metabolite **7** (27-321).

**Figure S25.** The HMBC of metabolite **7** (27-321).

**Figure S26.** The NOESY of metabolite **7** (27-321).

**Figure S27.** The ECD spectrum of metabolite **7**.

**Figure S28.** Chlorination of carviolin A (1) via Hal *in vitro*.

Table S2. The ^1^H and ^13^C NMR data of compounds **1**−**3** and **6**−**8**.

| No | **1**  **300** | **2**  **334** | **3**  **618 (purple)** | **6**  **288** | **7**  **322** | **8**  **367** |
| --- | --- | --- | --- | --- | --- | --- |
| 1 | 160.64 s | 161.03 s | 160.04 s / 159.97 s | 202.77 s | 203.69 s | 159.97 s |
| 2 | 116.26 d  7.50 s | 116.33 d  7.35 s | 109.82 d/ 113.20  7.15 s/ 7.03 s | 51.50 t  2.82 d, 17.2  2.75 d, 17.2 | 51.7 t  2.72 d, 16.9  2.64 d, 16.9 | 115.91  7.40 s |
| 3 | 151.45 s | 152.14s | 147.03 s/ 142.51s | 69.99 s | 69.96 s | 150.23 s |
| 4 | 116.81 d  7.76 s | 117.11 d  7.57 s | 124.52 d/ 127.69 d  7.31 s/ 7.17 s | 43.16 t  3.03 brd | 43.4 t  2.90 s | 117.04 d  7.67 s |
| 5 | 134.09 s | 134.68 s | 128.42 s/ 128.28 s | 142.19 s | 139.53 s | 136.23 s |
| 6 | 182.37 | 181.89 s | 127.76 s/ 127.72 s | 116.06 d  6.76 s | 112.18 d  7.06 s | 183.57 s |
| 7 | 134.73 s | 131.31 s | 138.54 s/ 138.45 s | 137.60 s | 137.50 s | 127.22 s |
| 8 | 108.28 d  7.06 | 106.60 d  7.09 s | 117.64 s/ 117.63 s | 102.25 d  6.61 s | 105.02 s | 120.94 s |
| 9 | 164.24 s | 156.53 s | 165.06 s/ 165.06s | 160.33 s | 156.00 s | 166.33 s |
| 10 | 107.06 d  6.58 s | 113.52 s | 101.72 s/ 101.63 s | 97.63 d  6.49 s | 97.75 d  6.58 s | 112.70 s |
| 11 | 164.51 s | 160.20 s | 166.63 s/ 166.74 s | 161.87 s | 159.95 s | 160.91 s |
| 12 | 110.18 s | 110.48 s | 109.23 s/ 109.22 s | 109.21 s | 109.40 s | 104.09 s |
| 13 | 186.22 s | 186.44 s | 183.50 s/ 183.50 s | 165.93 s | 165.15 s | 182.56 s |
| 14 | 118.37 s | 118.19 s | 117.74 s/ 116.91 s | 109.25 s | 109.47 s | 119.16 s |
| 15 | 62.25 t  4.65 s | 62.67 t  4.59 s | 62.74 t/ 22.02 q  4.31 s/ 2.12 s | 28.39 q  1.37 s | 29.41 q  1.28 s | 62.83 t  4.63 s |
| 16 | 56.41 q  3.95 s | 56.70 q  3.88 s | 56.51 q/56.51 q  4.00 s/3.98 s | 55.31 q  3.92 s | 56.11 q  3.86 s | 56.76 q  3.91 s |
| 11-OH | 13.27 s | 13.85 s |  |  |  | 13.85 s |
| 13-OH |  |  |  | 15.06 s | 14.83 s |  |

**Table S3** **Amplification of sgRNA and validation primers.**

| **Primer name** | **Sequence (5' to 3')** | **PAM** |
| --- | --- | --- |
| *hal*sg-F | ATAGCCATTGGGGCCACCCGgttttagagctagaaatagcaagttaaaat | CGG |
| *hal*sg-R | CGGGTGGCCCCAATGGCTATTGCATGATCCGCGAATCG |  |
| *AnOsg-*F | TCTGGCCGCAAGTATCTCTGGTTTTAGAGCTAGAAATAGCAAGTTAAAAT | TGG |
| *AnOsg-*R | CAGAGATACTTGCGGCCAGATGCATGATCCGCGAATCG |  |
| Y*hal*-F | ATGCTCCCTTCCATGCG |  |
| Y*hal*-R | AGCACCCTCGTAGTGGTG |  |
| Y*anO-*F | GGCGCAGTTTCGTCACATT |  |
| Y*anO-*R | GTCCCCTTTTCGGTCGGTTC |  |


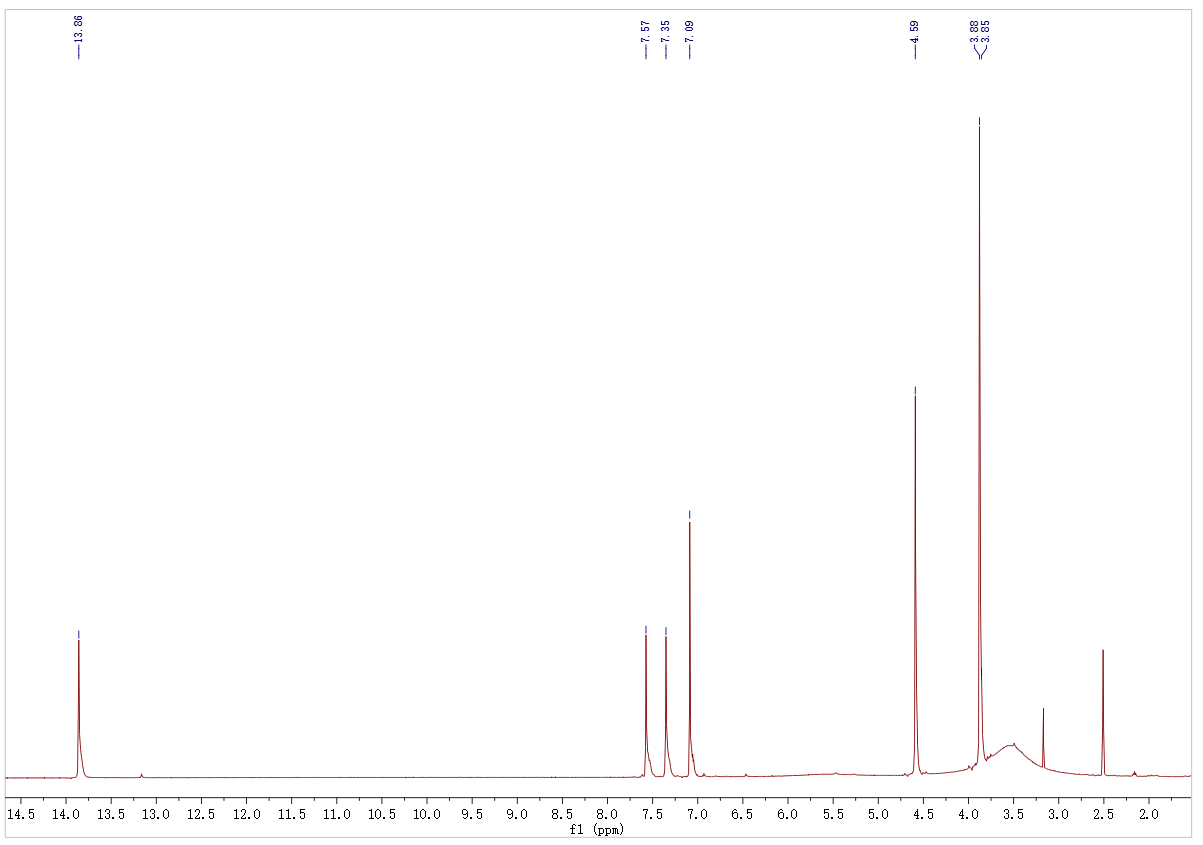


Figure S1. The ^1^H NMR of metabolite **2** (27-334).


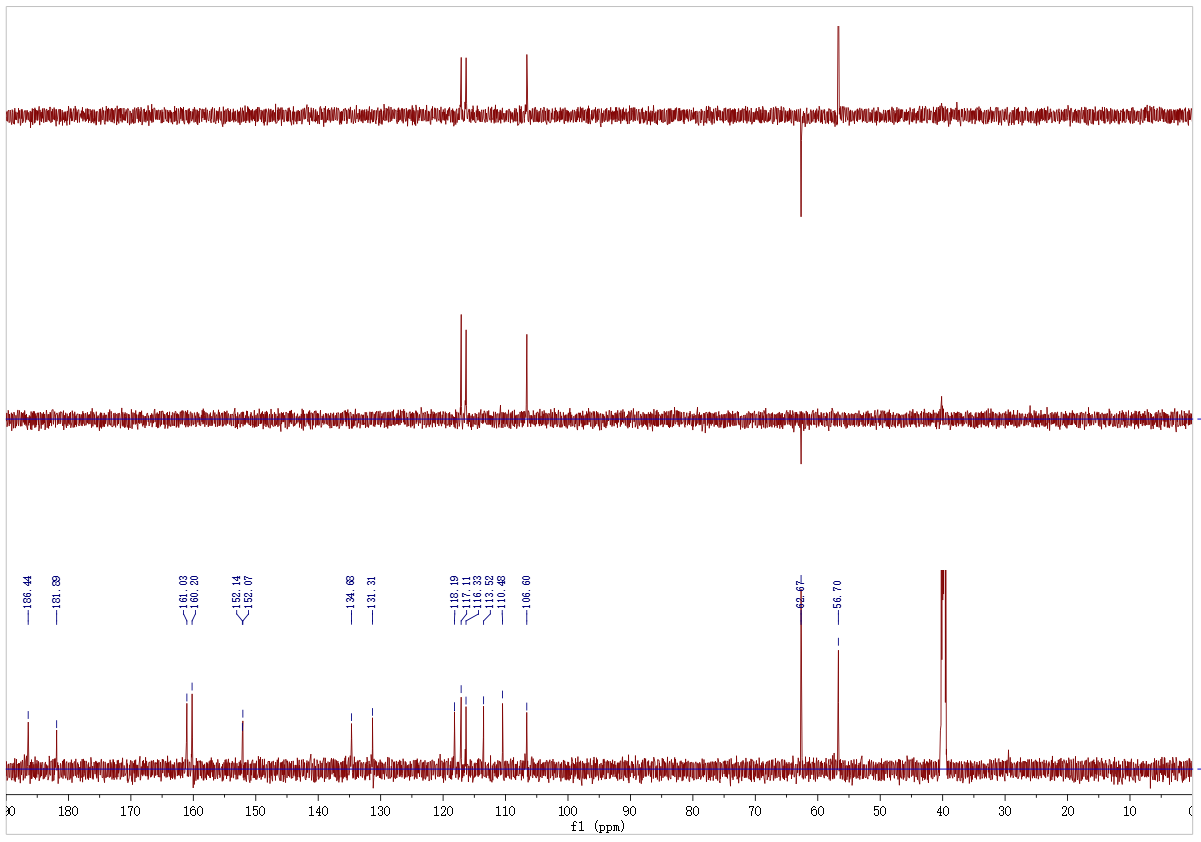


Figure S2. The ^13^C NMR of metabolite **2** (27-334).


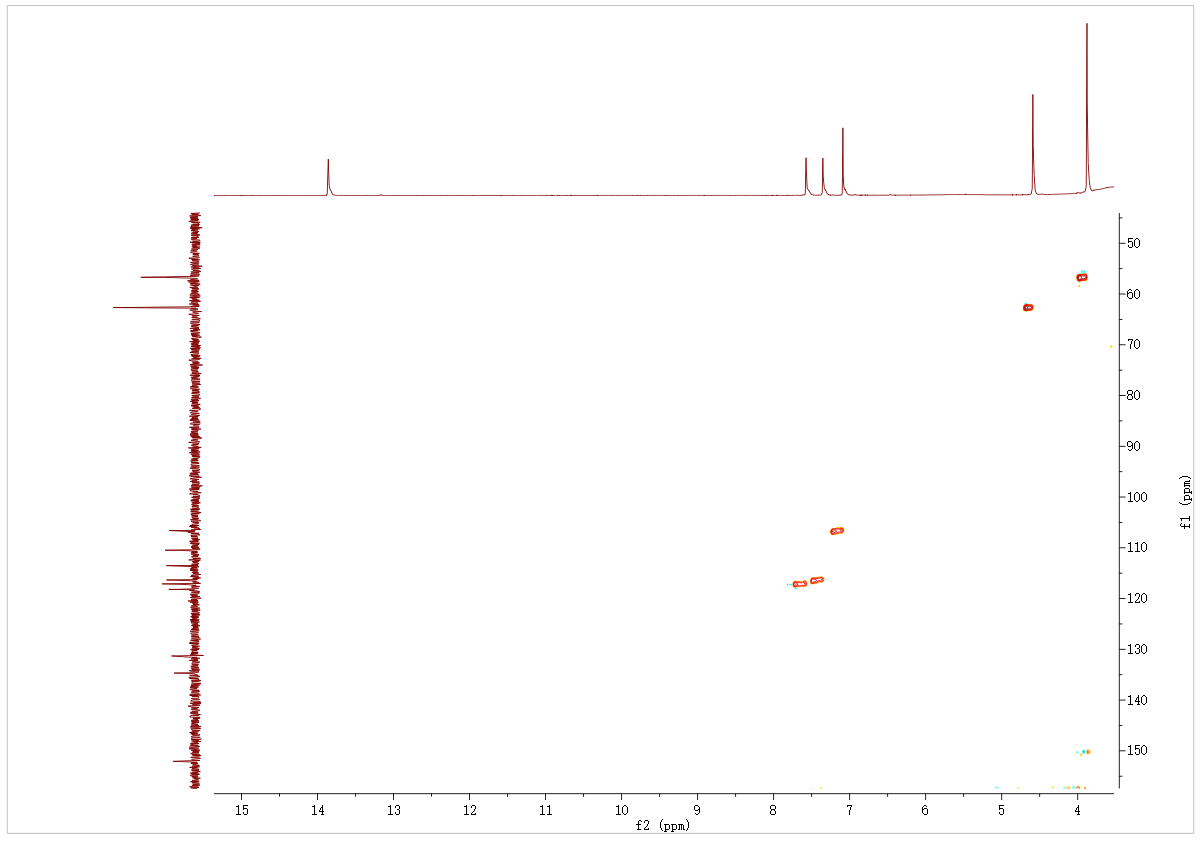


Figure S3. The HSQC of metabolite **2** (27-334).


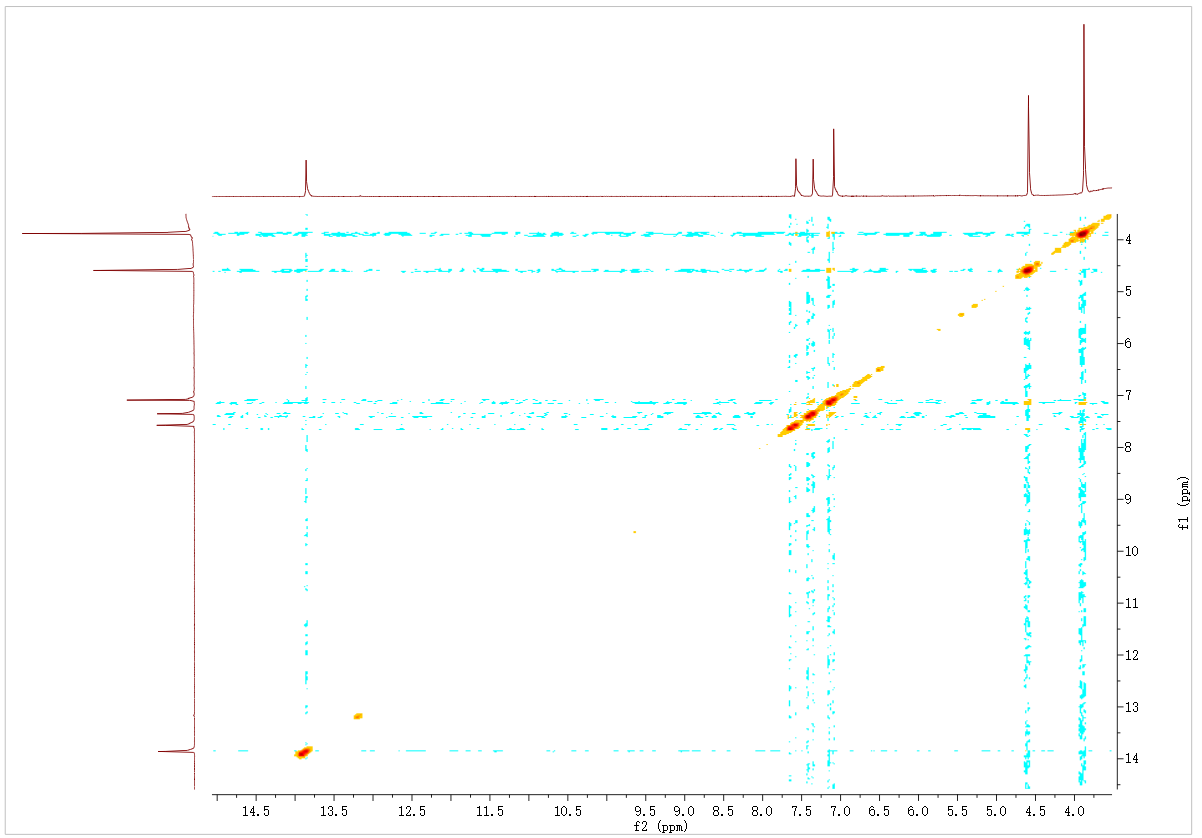


Figure S4. The COSY of metabolite **2** (27-334).


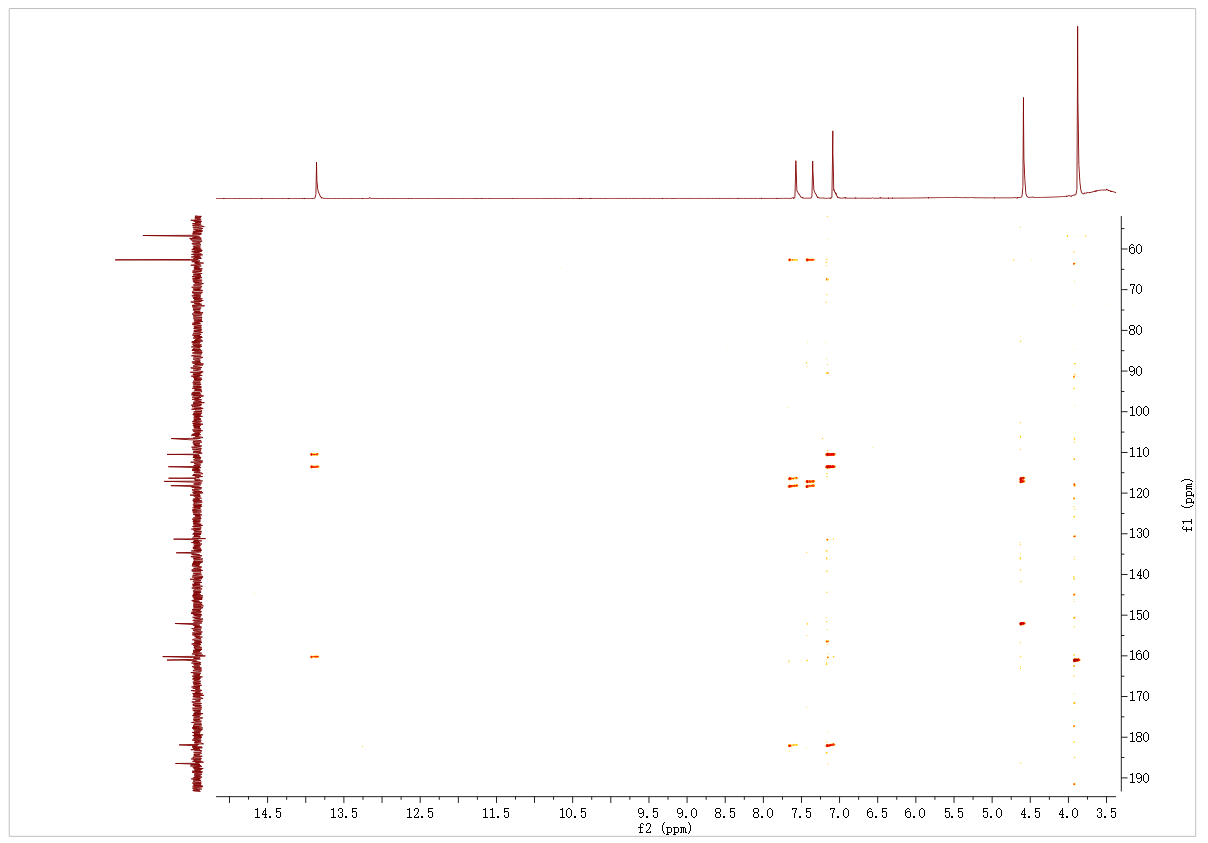


Figure S5. The HMBC of metabolite **2** (27-334).


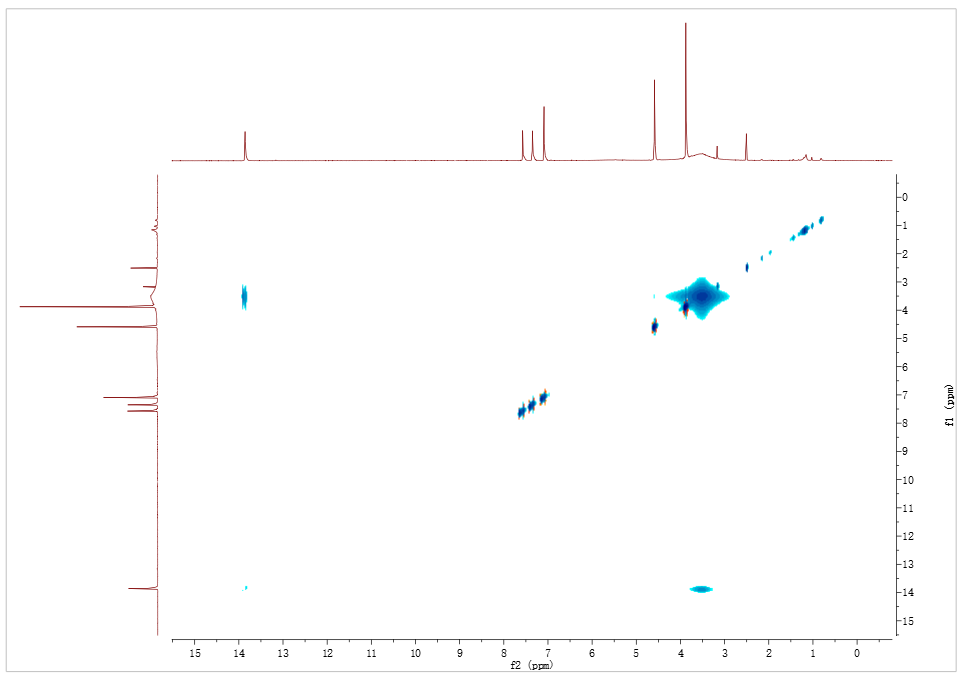


Figure S6. The NOESY of metabolite **2** (27-334).


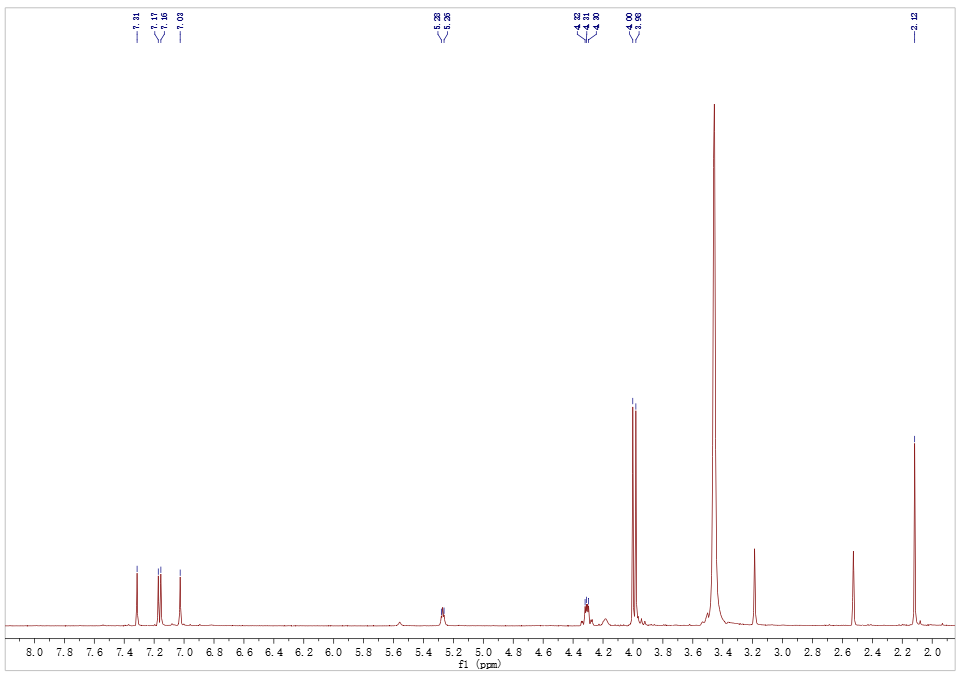


Figure S7. The ^1^H NMR of metabolite **3** (purple).


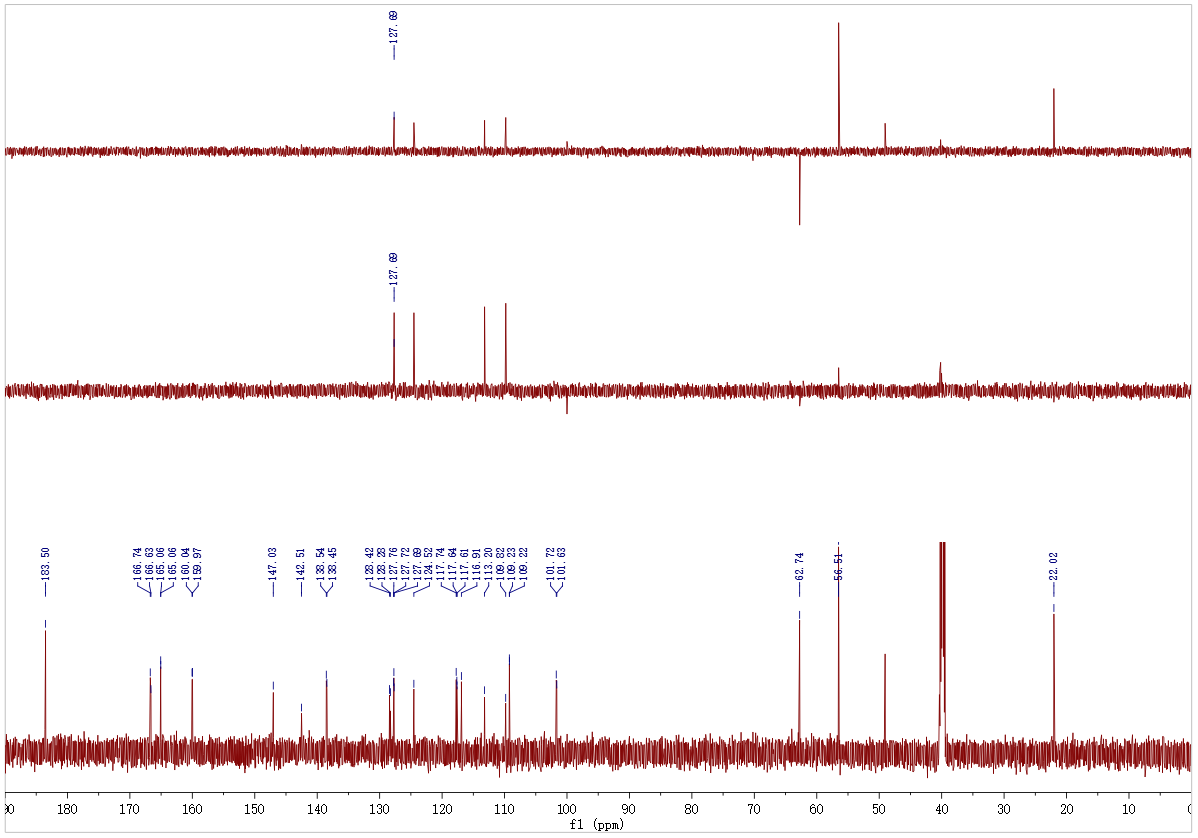


Figure S8. The ^13^C NMR of metabolite **3** (purple).


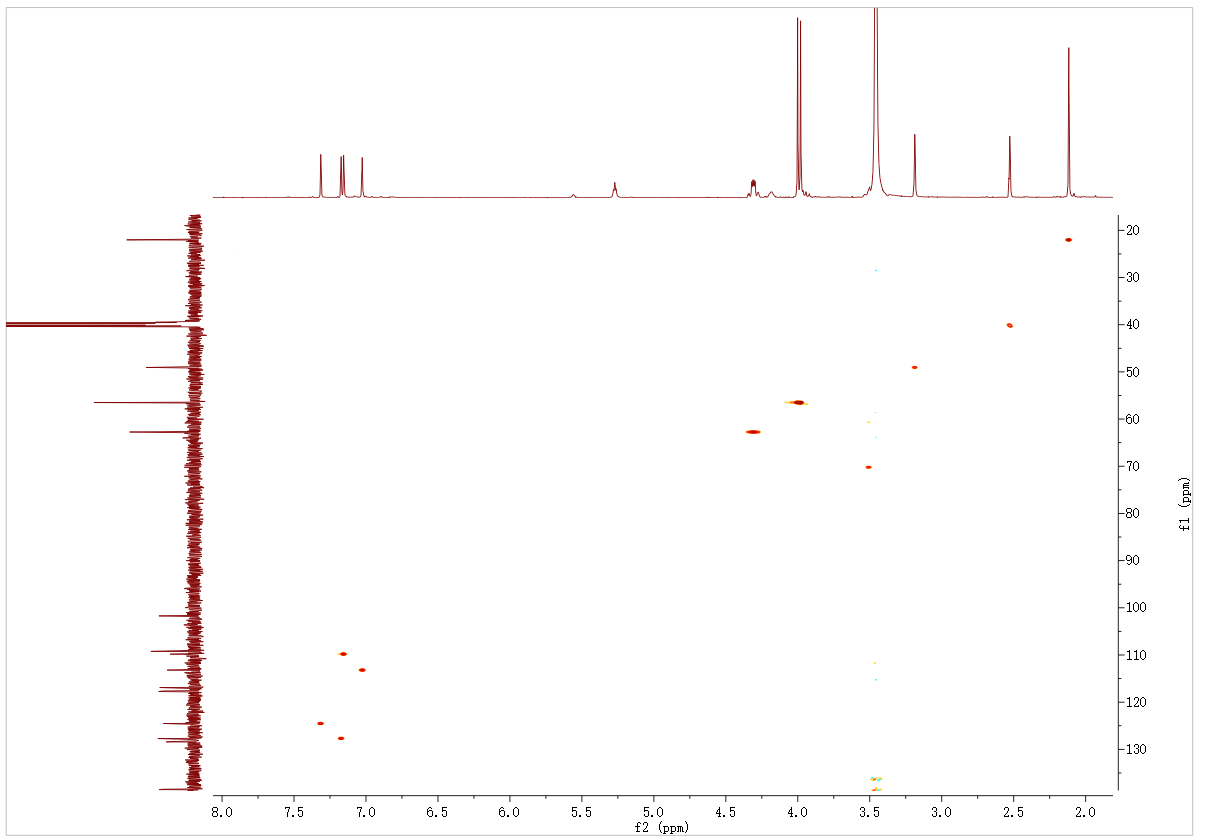


Figure S9. The HSQC spectrum of metabolite **3** (purple).


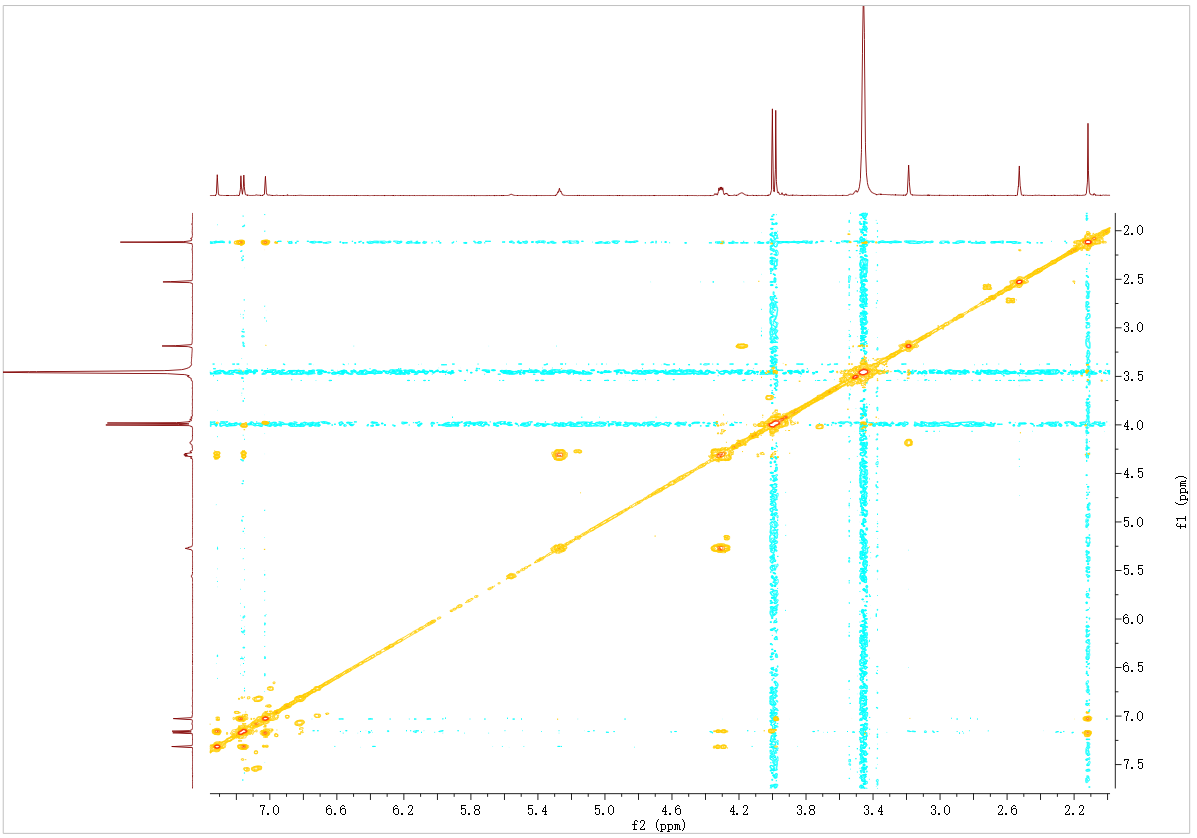


Figure S10. The COSY spectrum of metabolite **3** (purple).


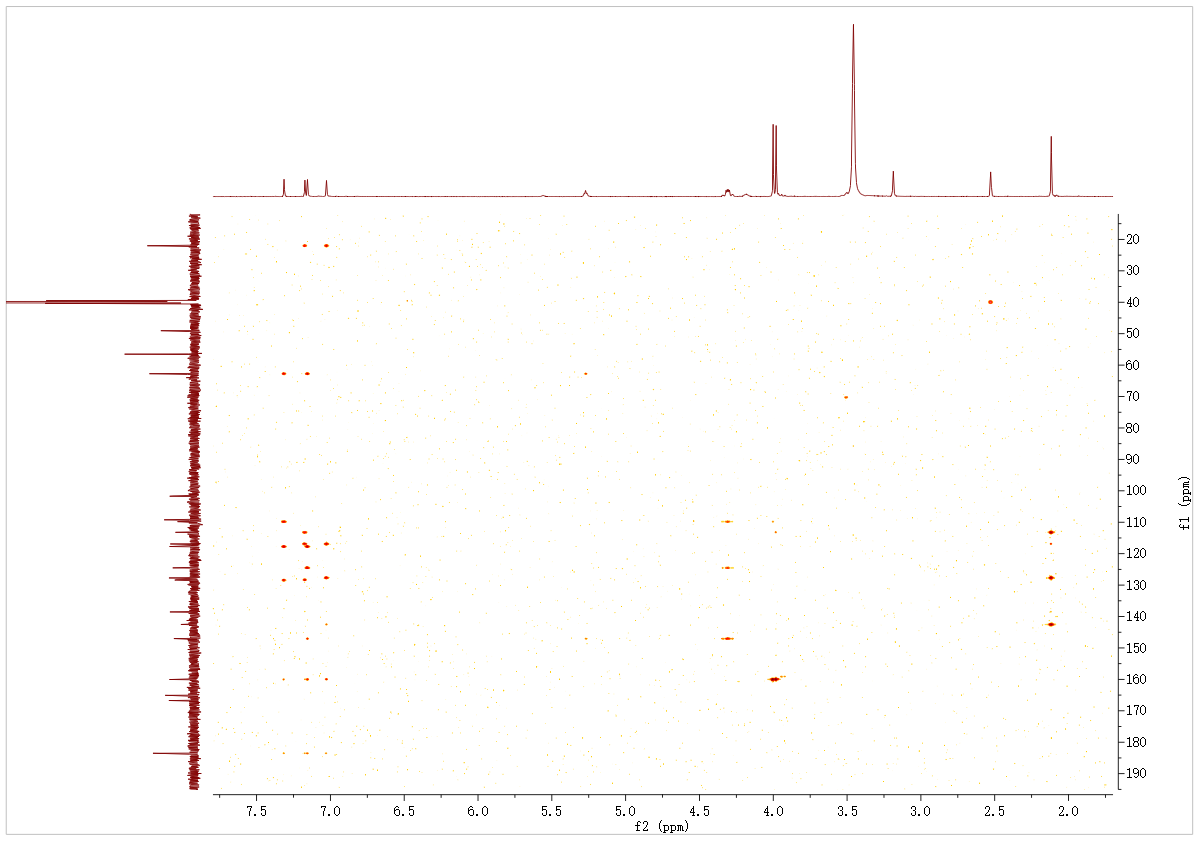


Figure S11. The HMBC spectrum of metabolite **3** (purple).


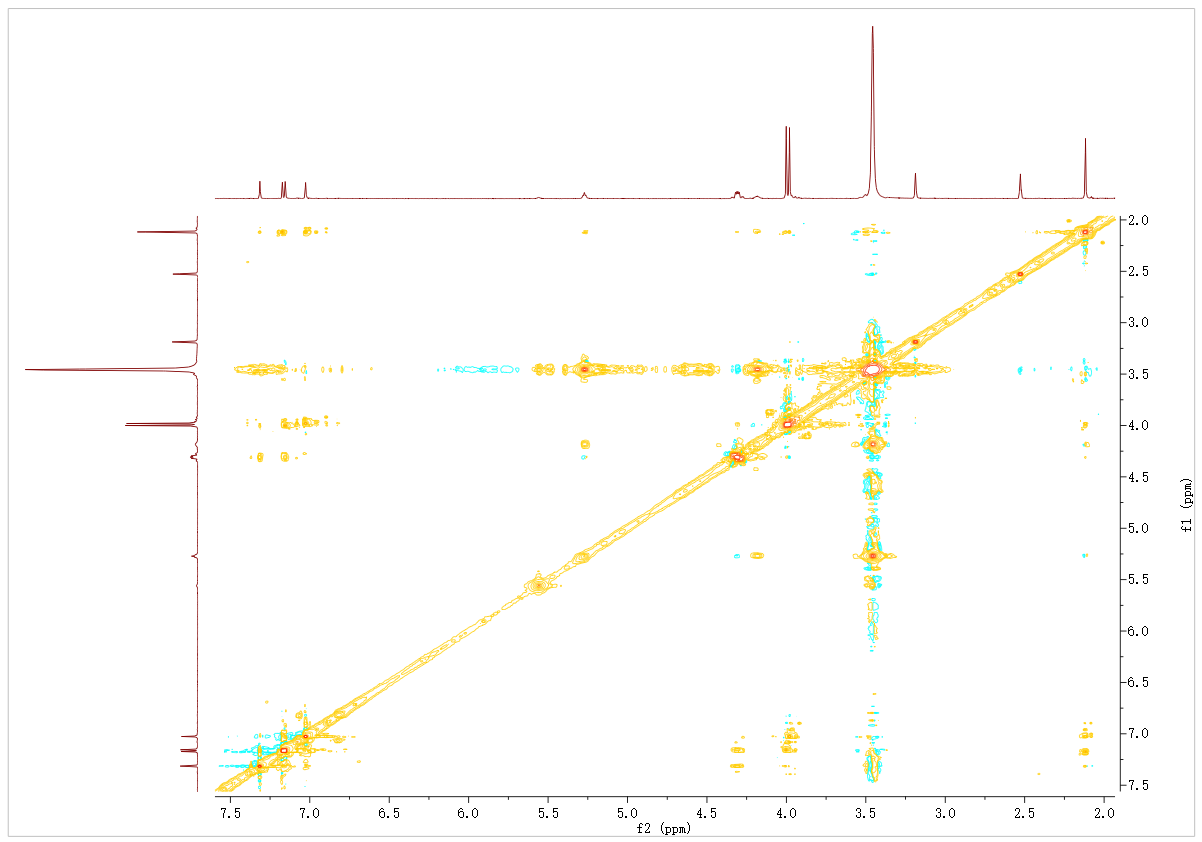


Figure S12. The NOESY spectrum of metabolite **3** (purple).


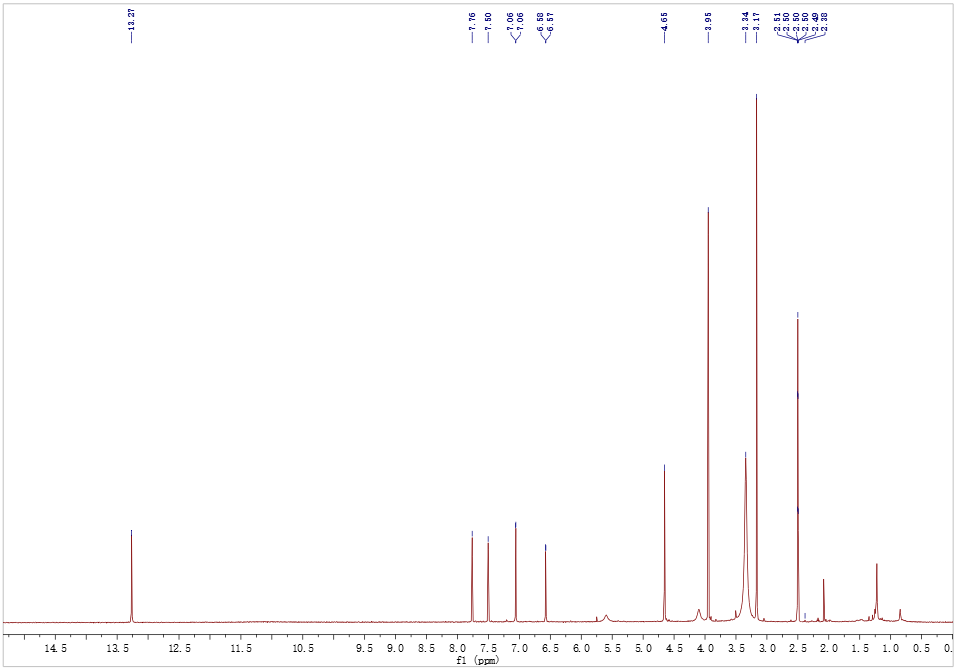


Figure S13. The ^1^H NMR of CA (**1**).


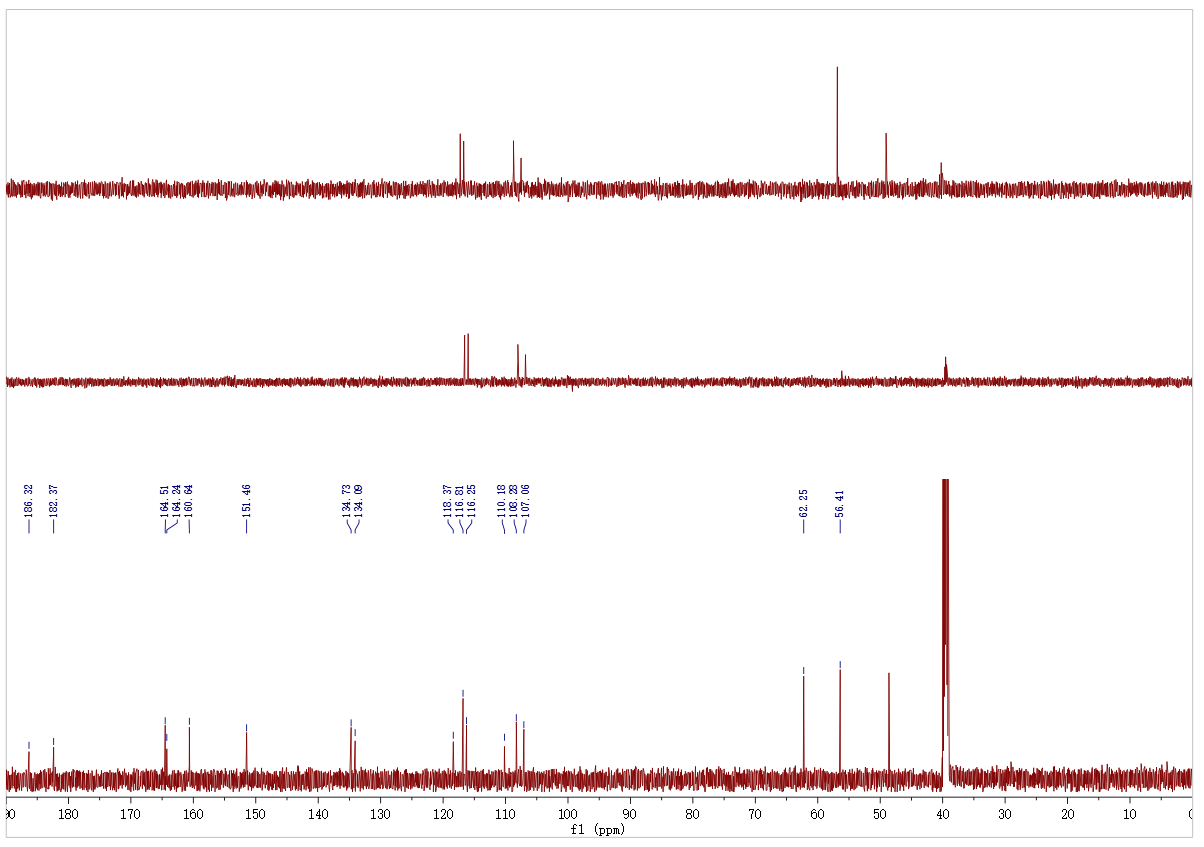


Figure S14. The ^13^C NMR of CA (**1**).


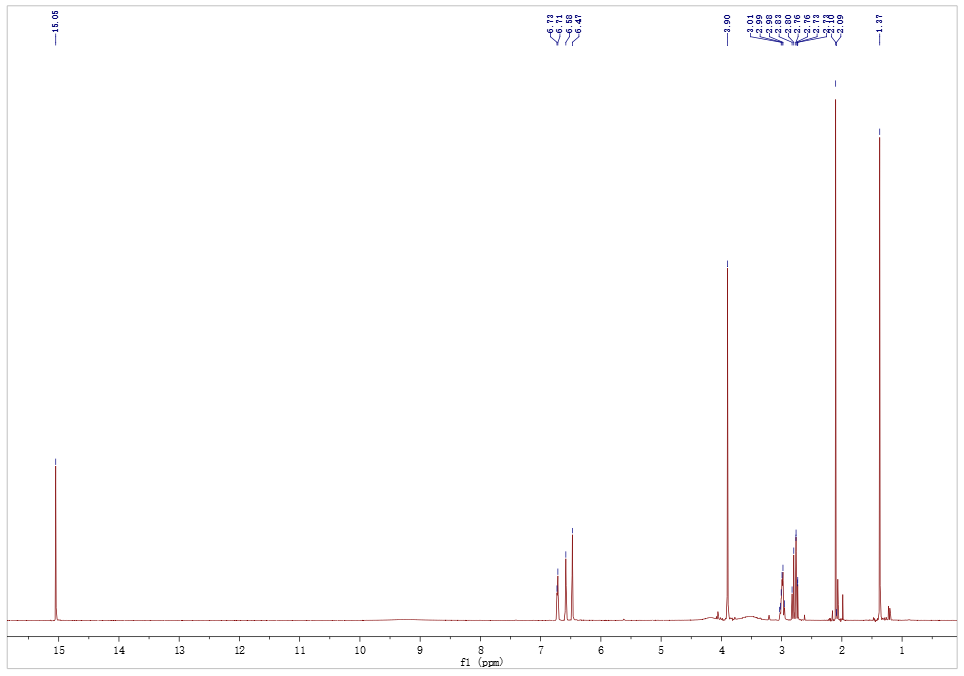


Figure S15. The ^1^H NMR of metabolite **6** (27-288).


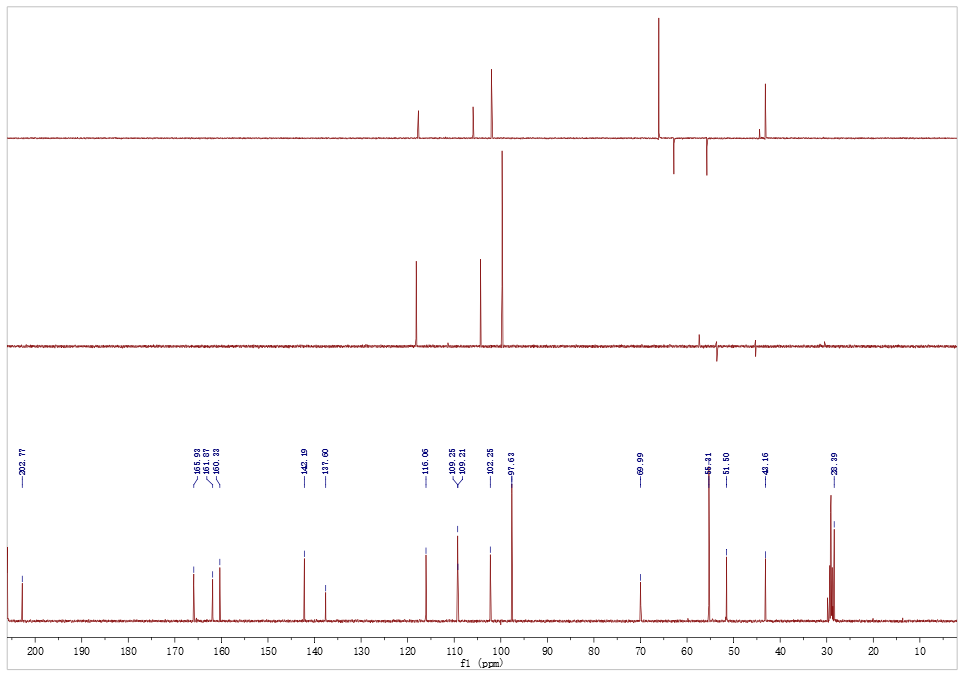


Figure S16. The ^13^C NMR of metabolite **6** (27-288).


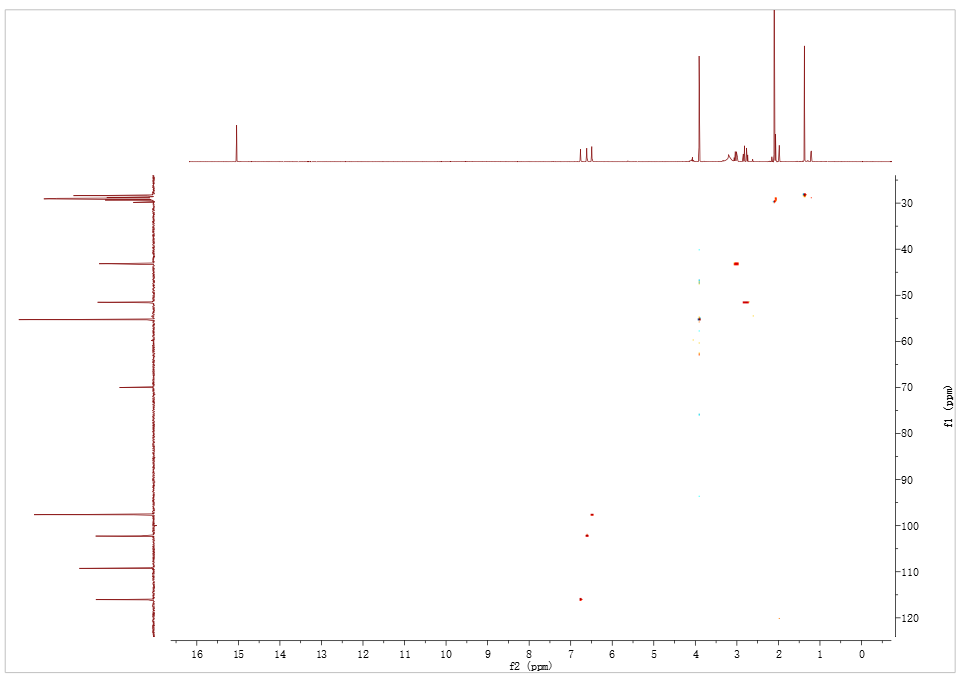


Figure S17. The HSQC of metabolite **6** (27-288).


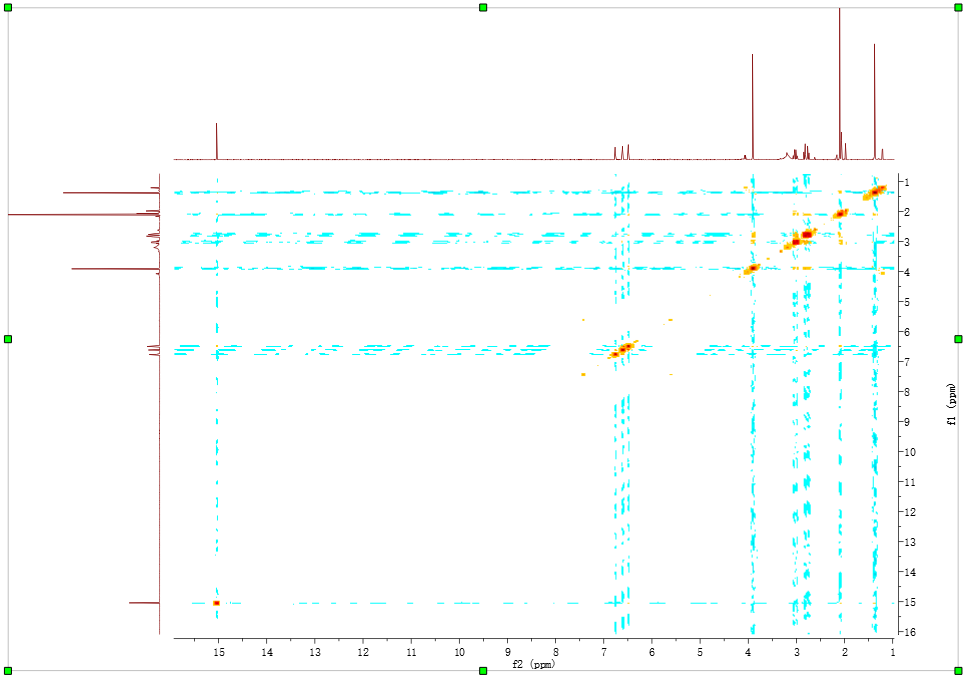


Figure S18. The COSY of metabolite **6** (27-288).


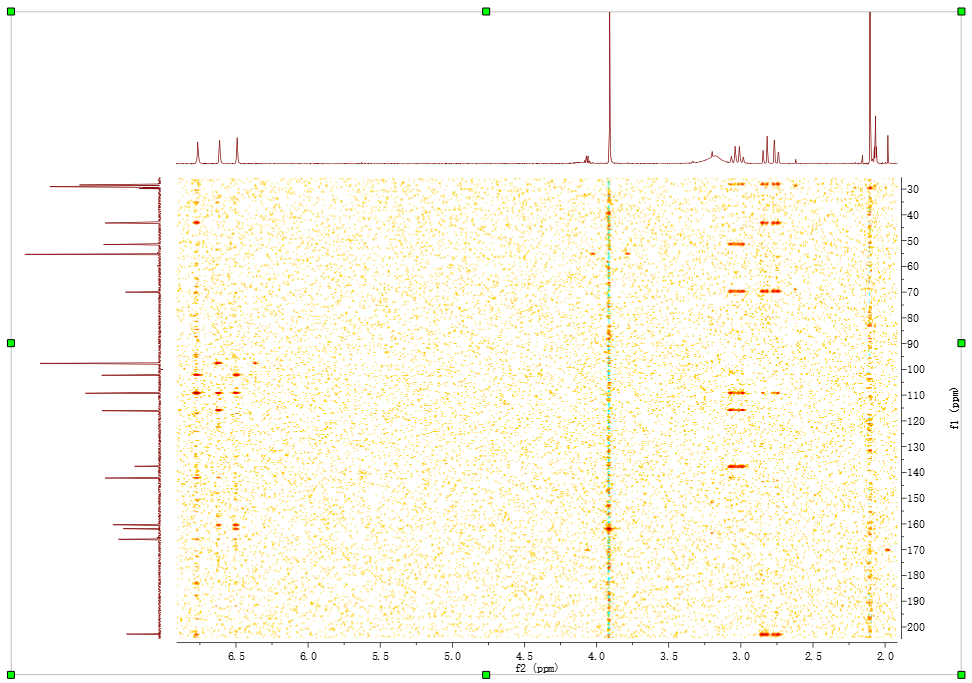


Figure S19. The HMBC of metabolite **6** (27-288).


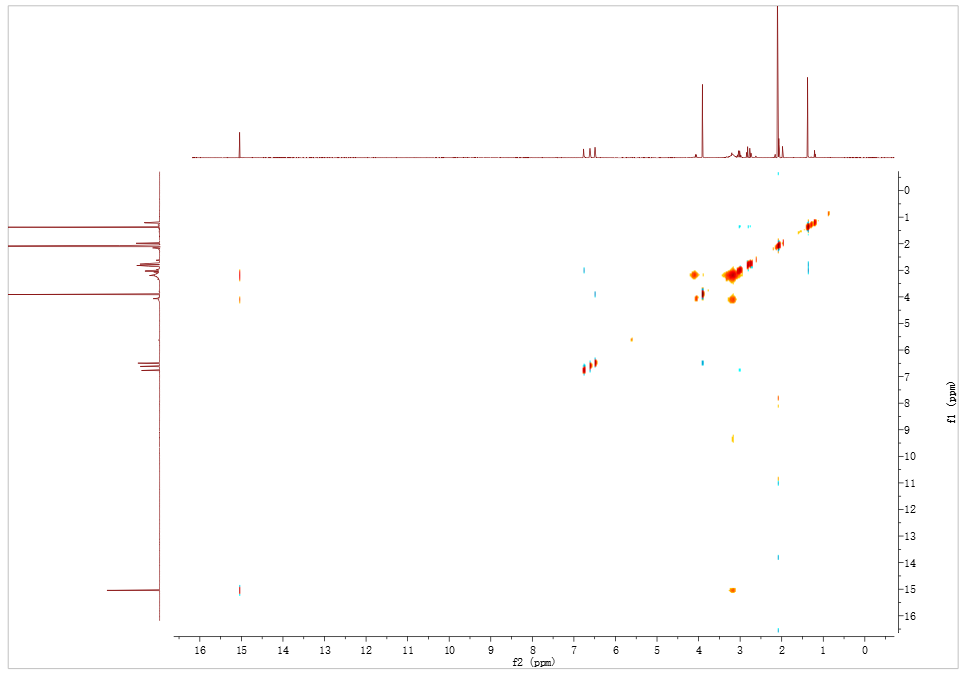


Figure S20. The NOESY of metabolite **6** (27-288).


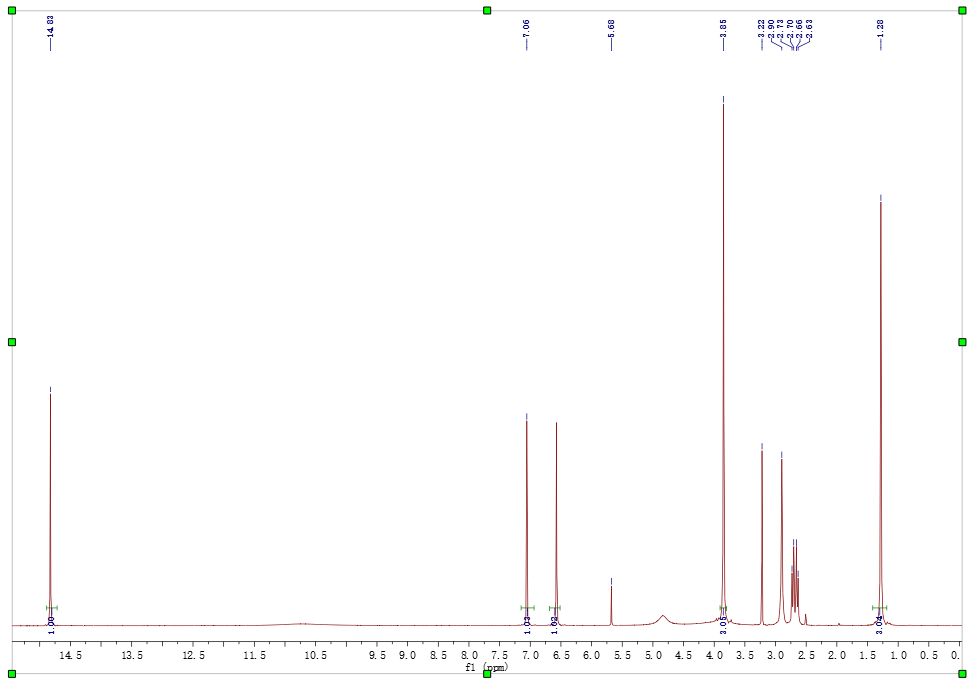


Figure S21 . The ^1^H NMR of metabolite **7** (27-321).


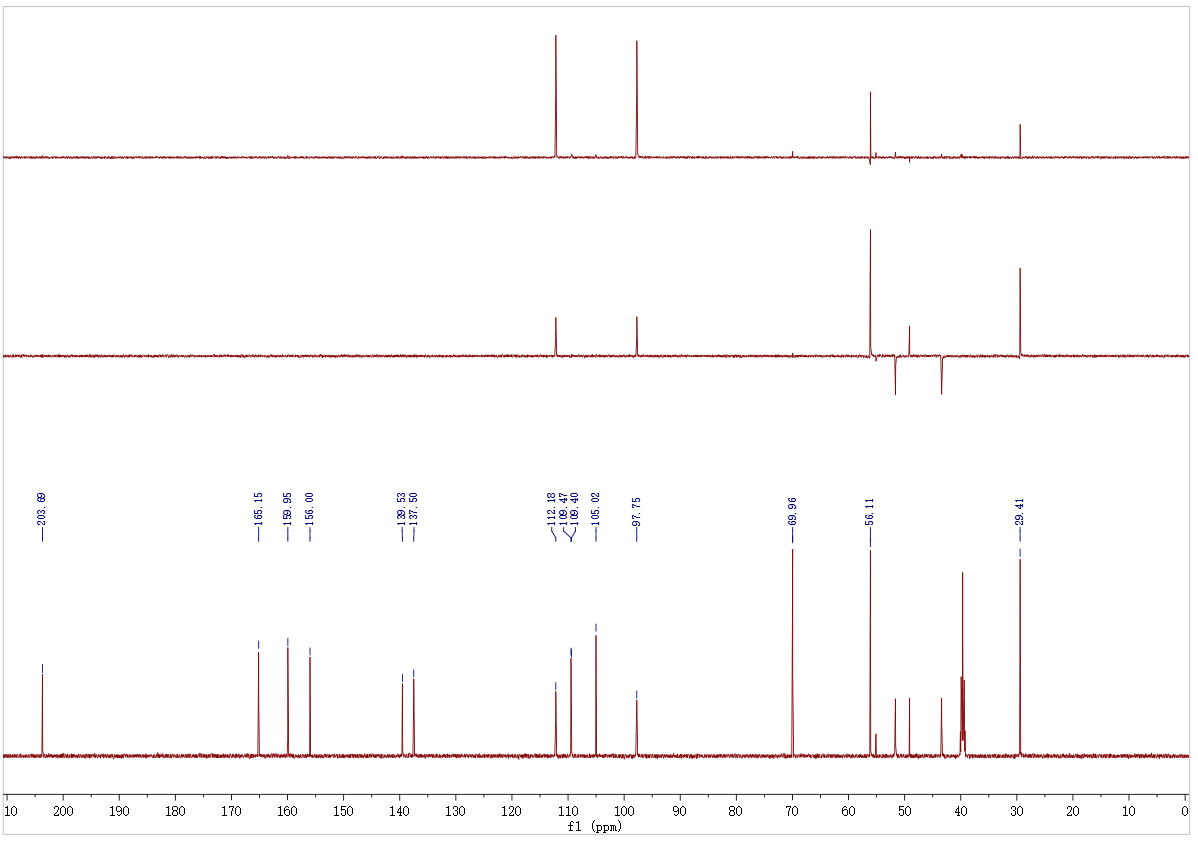


Figure S22. The ^13^C NMR of metabolite **7** (27-321).


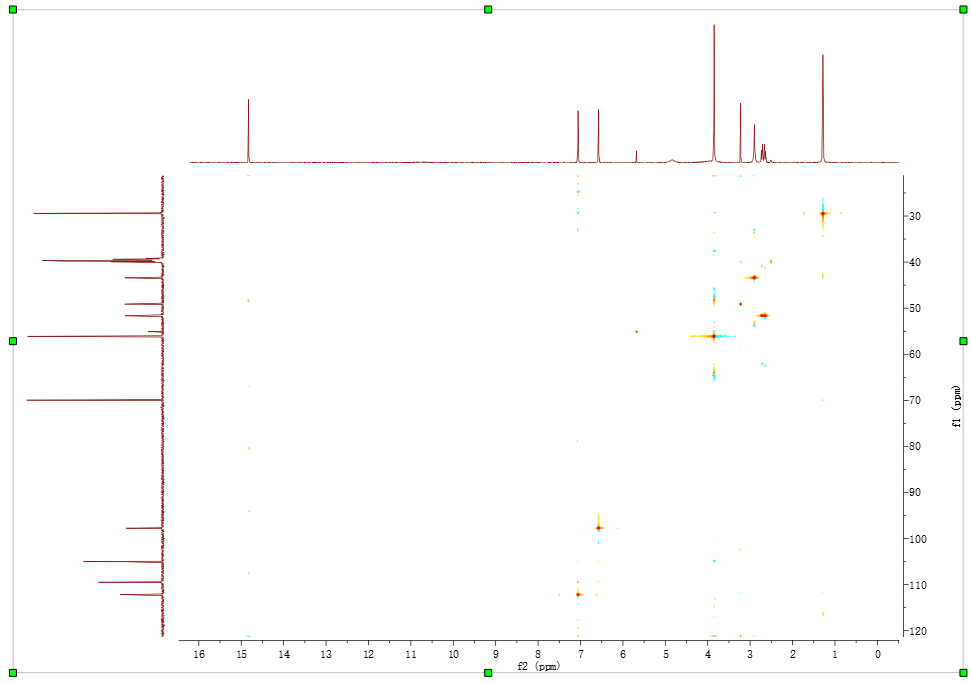


Figure S23. The HSQC of metabolite **7** (27-321).


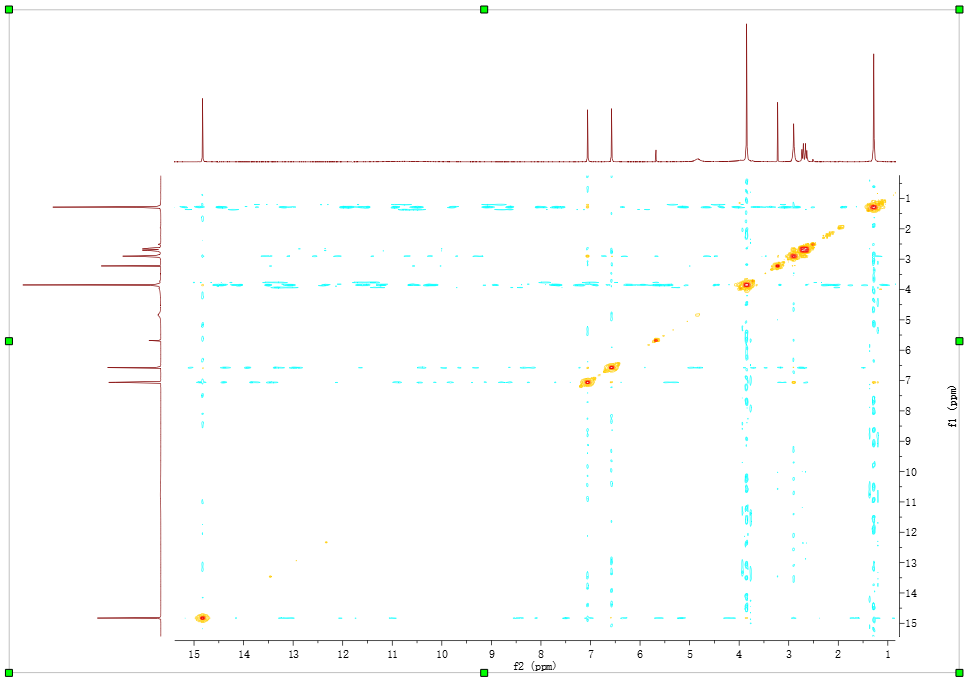


Figure S24. The COSY of metabolite **7** (27-321).


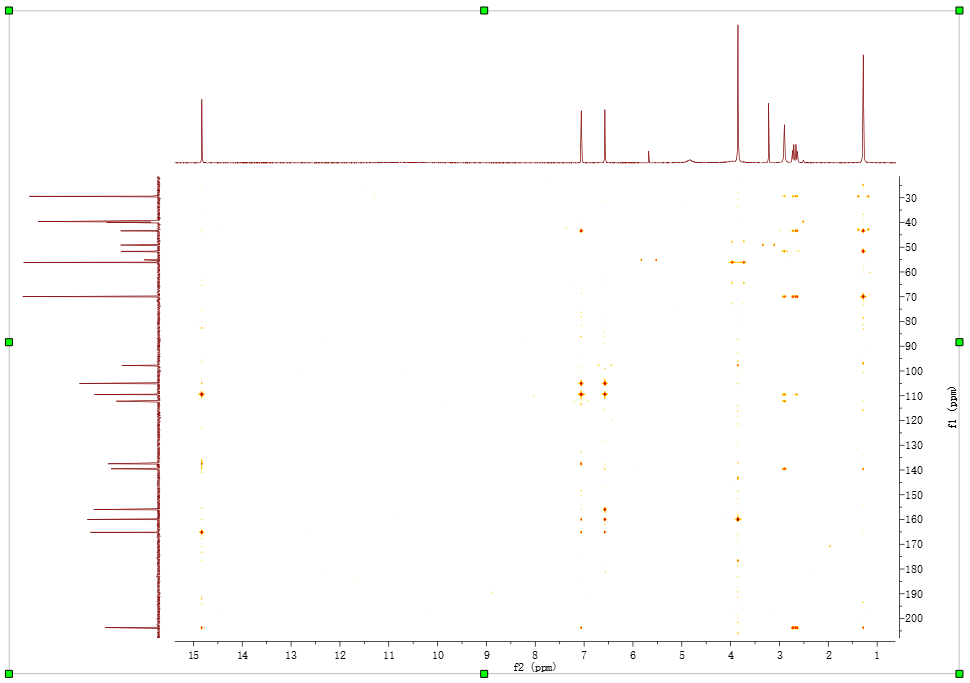


Figure S25. The HMBC of metabolite **7** (27-321).


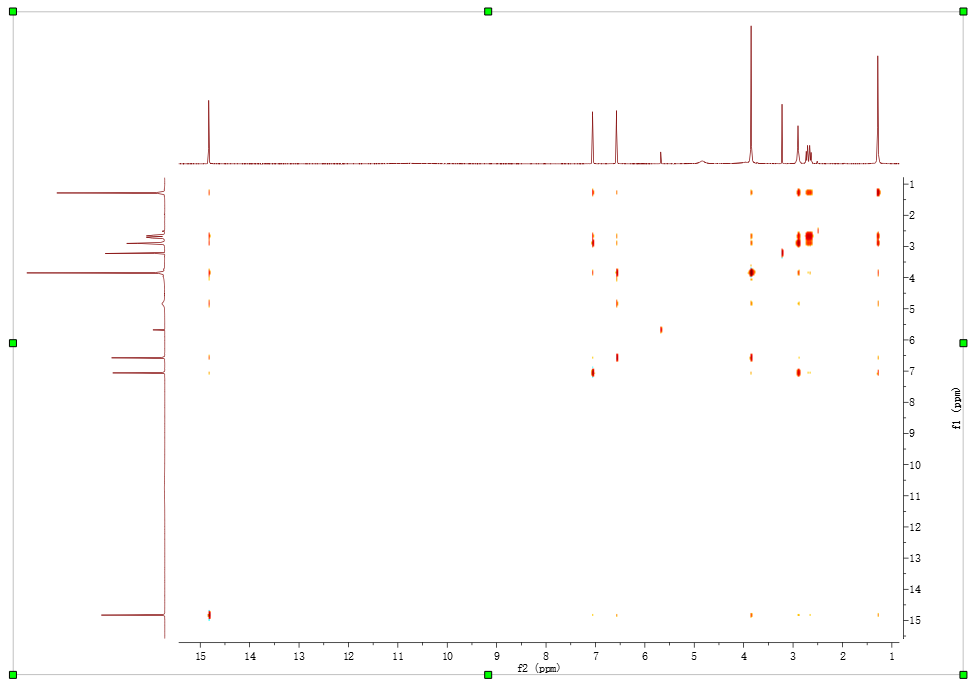


Figure S26. The NOESY of metabolite **7** (27-321).


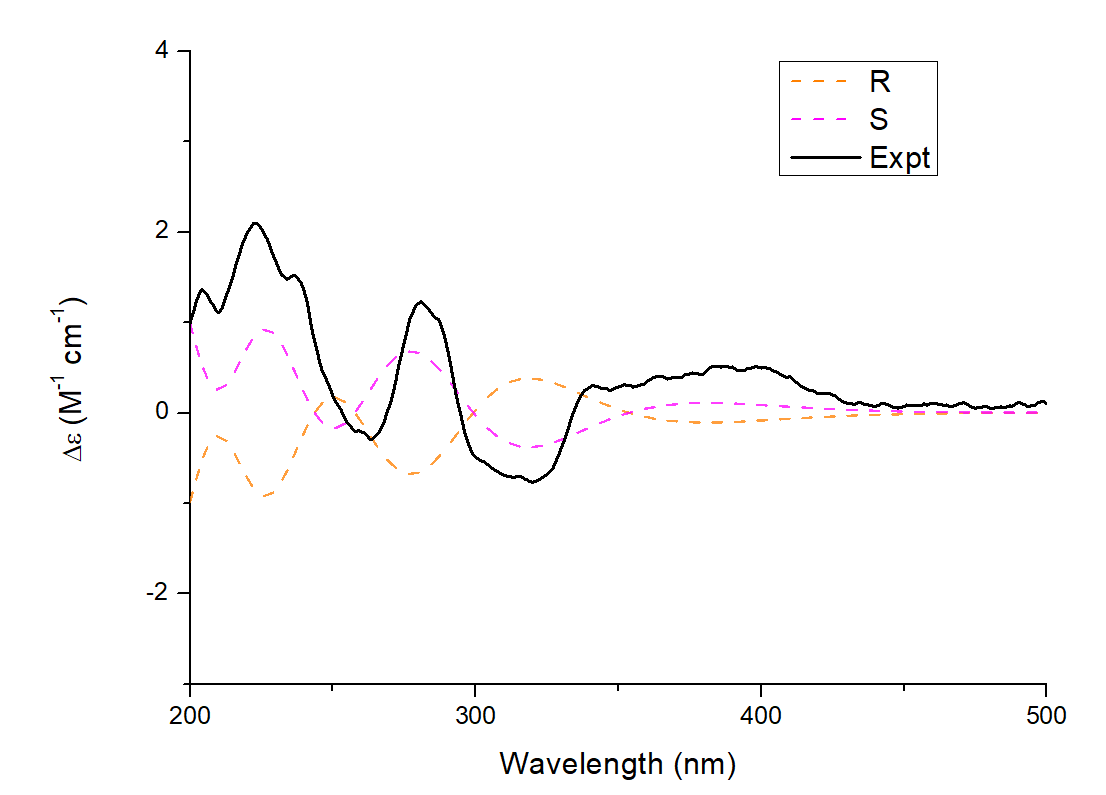


Figure S27. The ECD spectrum of metabolite **7**.


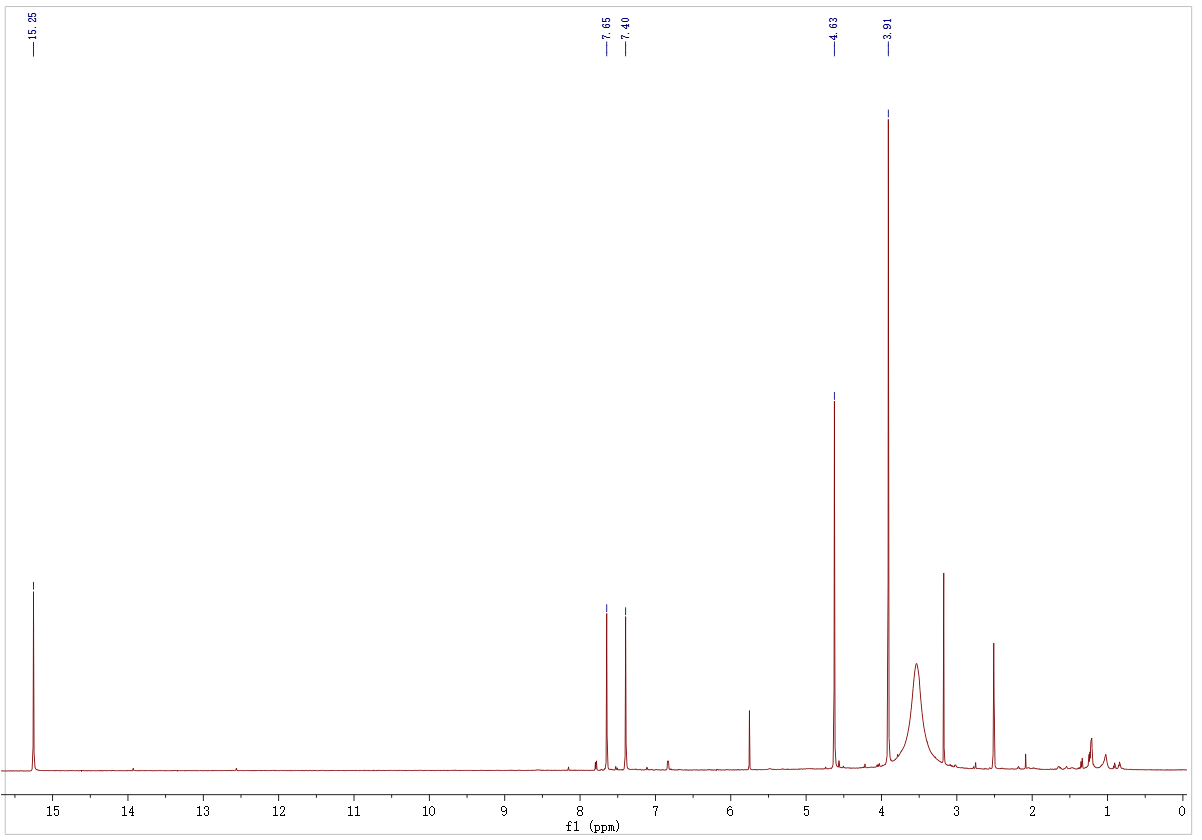


Figure S28. The ^1^H NMR spectrum of metabolite **8** (367).


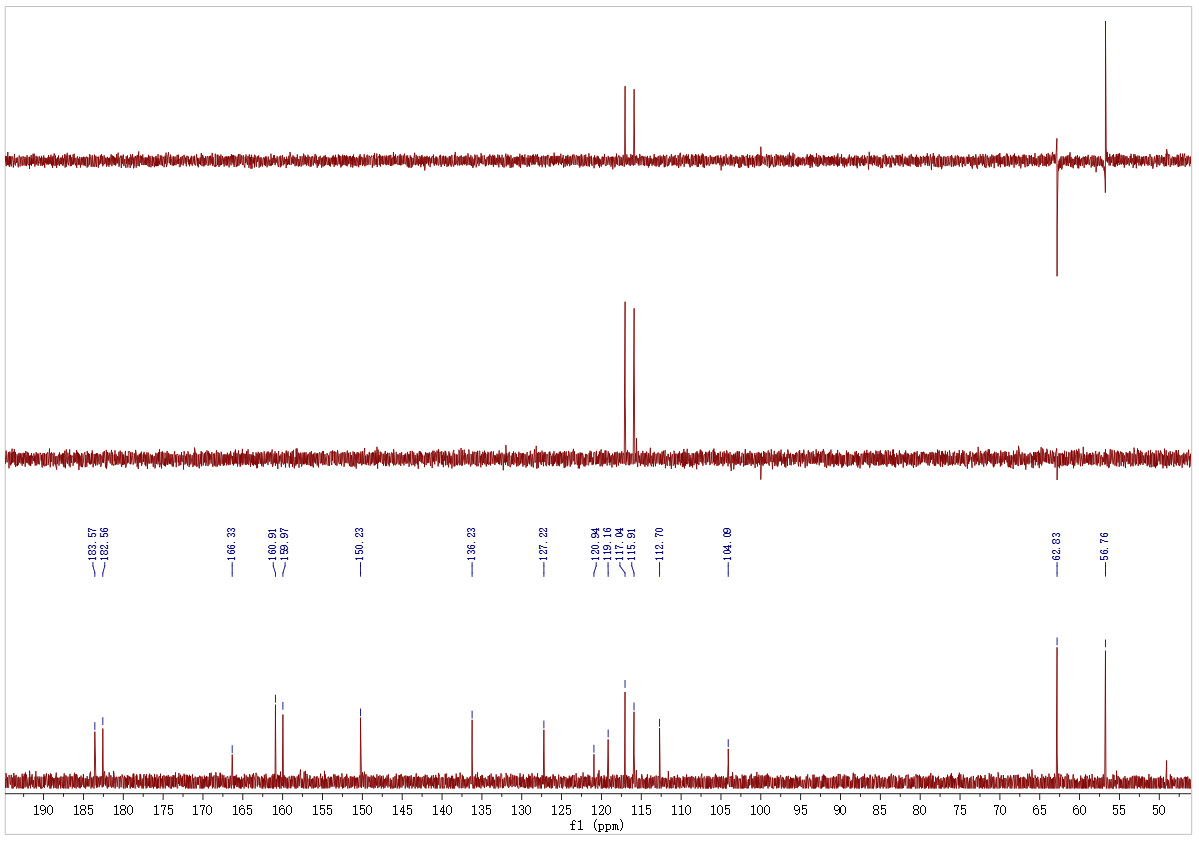


Figure S29. The ^13^C NMR spectrum of metabolite 8 (367).


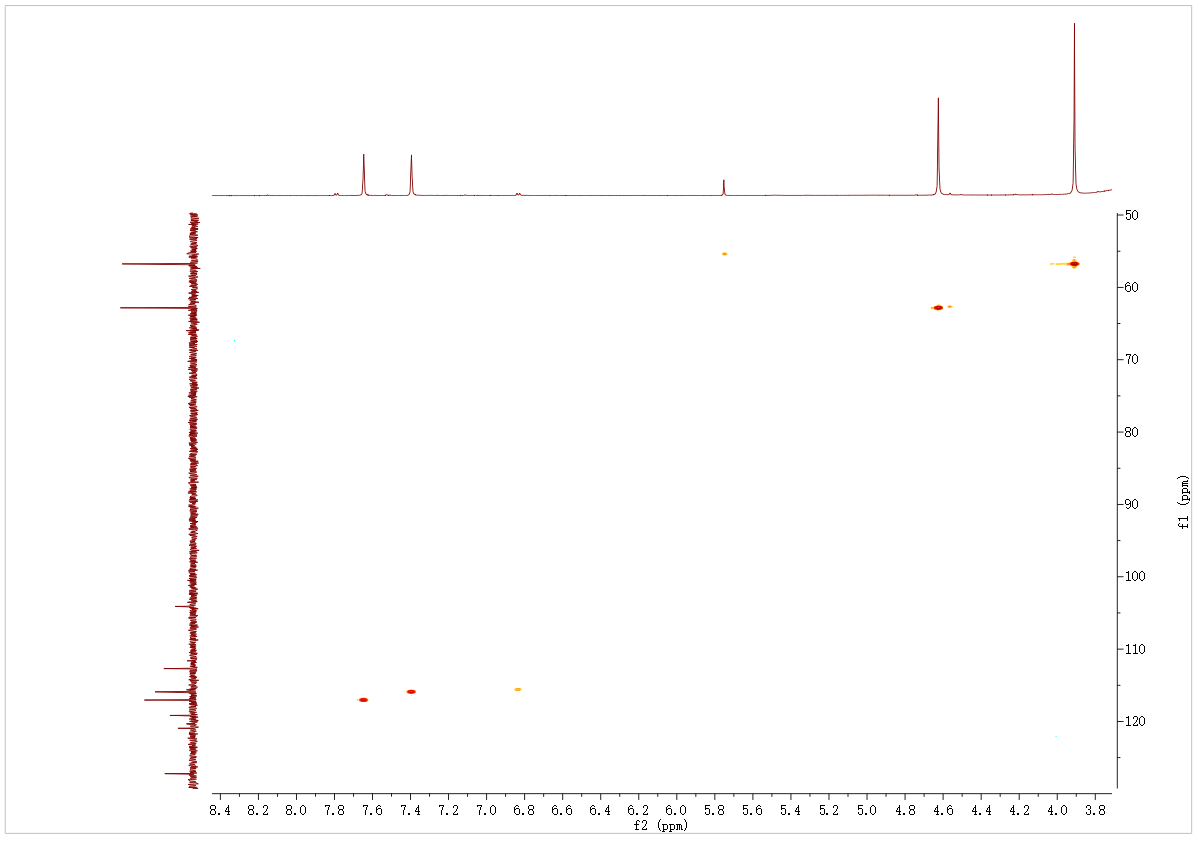


Figure S30. The HSQC spectrum of metabolite **8** (367).


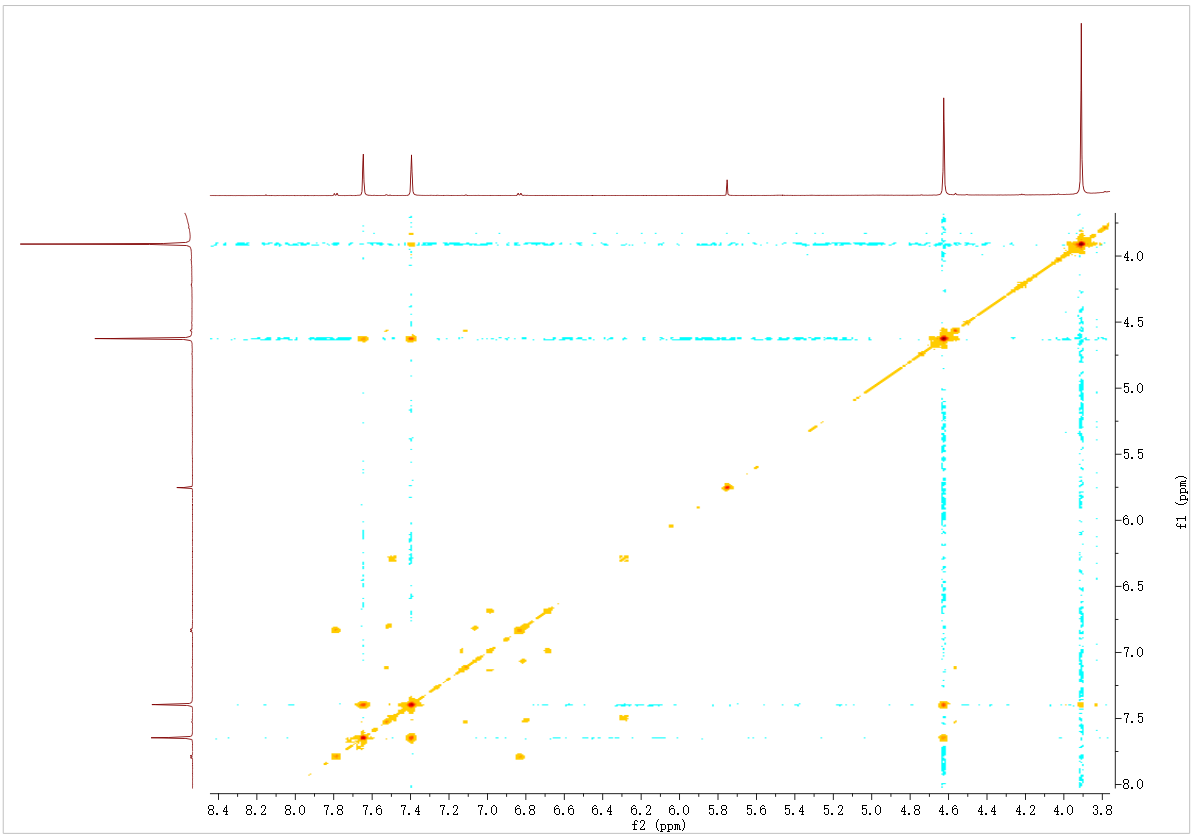


Figure S31. The COSY spectrum of metabolite **8** (367).


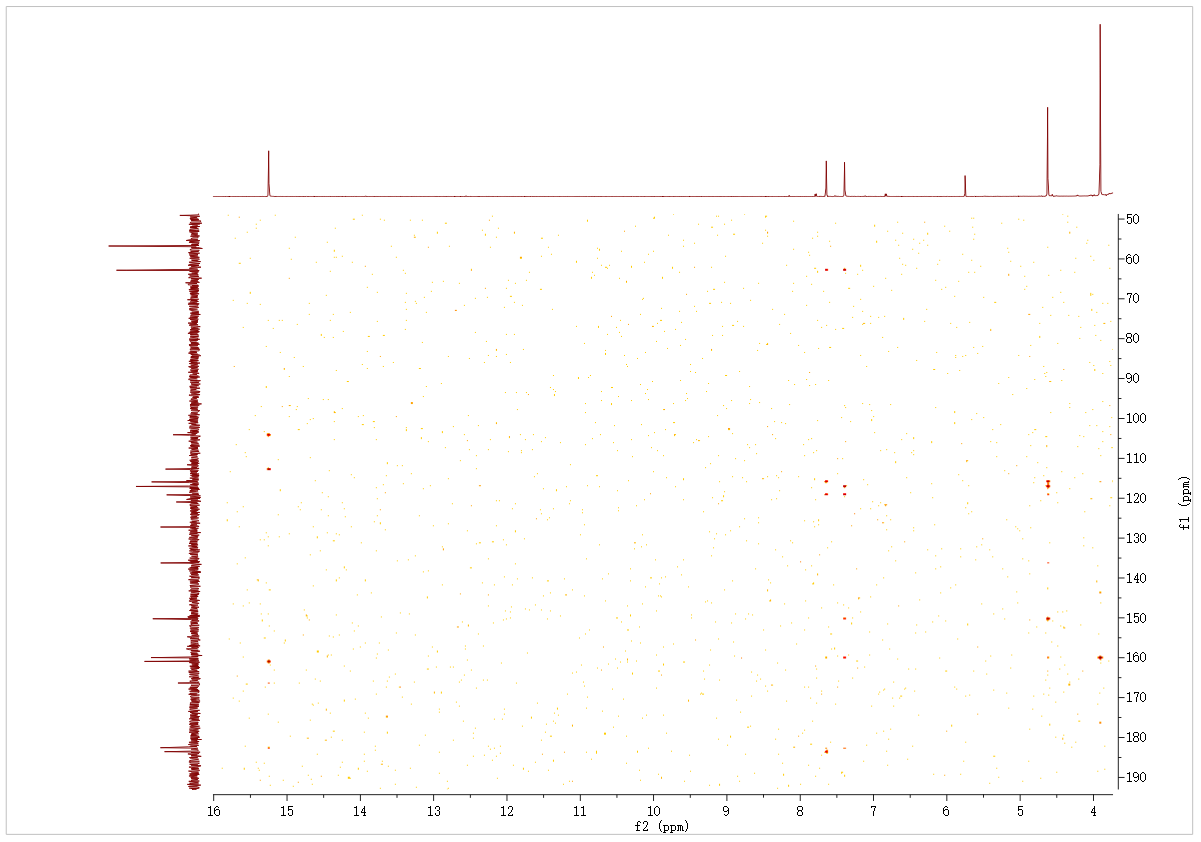


Figure S32. The HMBC spectrum of metabolite **8** (367).


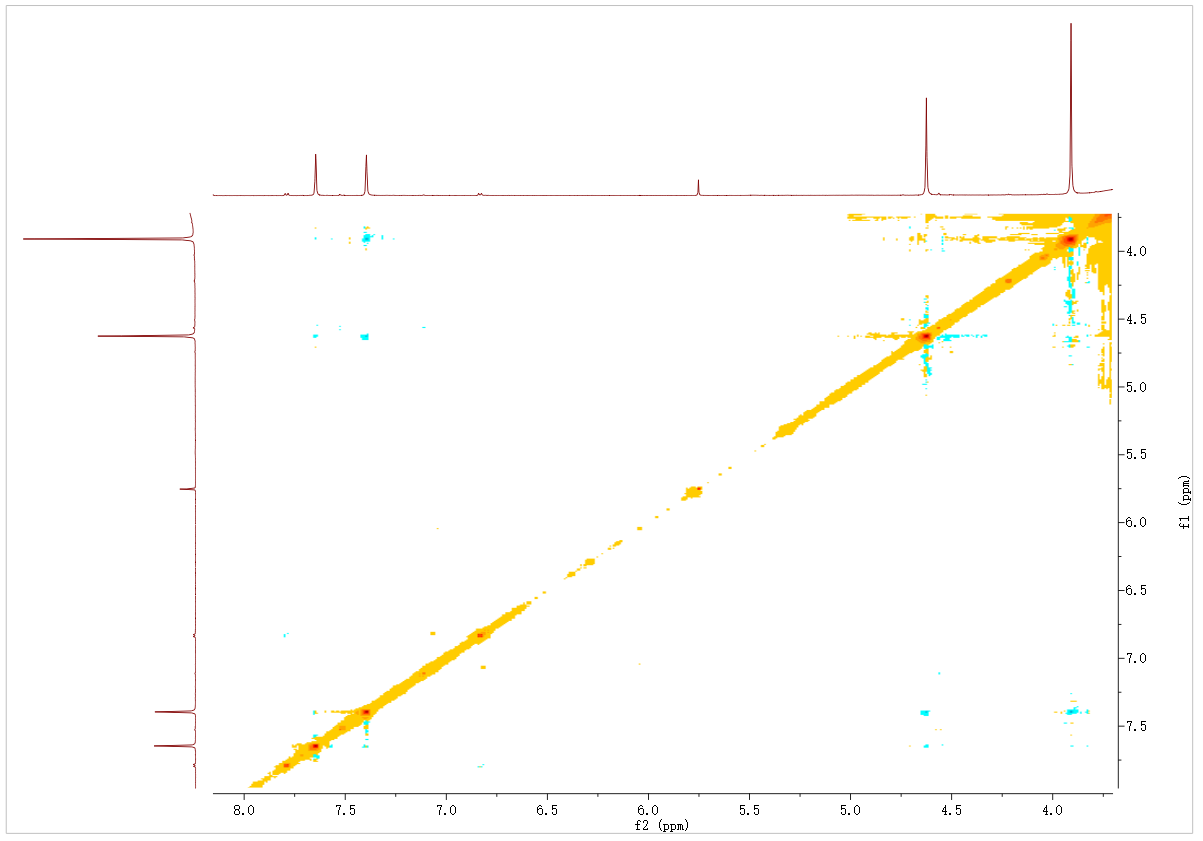


Figure S33. The NOESY spectrum of metabolite **8** (367).

**Fig. S34.** A) PCR analysis of gene *hal*. B) Isolation and purification of the protein Hal from *hal-*containing *E. coli* BL 21. C−D) HPLC-PDA/MS analysis displayed the chlorination of carviolin A (1) via Hal *in vitro*.
